# Cortical Somatostatin Neurons Regulate Seizure Susceptibility via MINAR1/Gαs–cAMP Signaling

**DOI:** 10.1002/advs.202519388

**Published:** 2026-02-04

**Authors:** Wei‐Tang Liu, Zhi‐Bin Hu, Ling Hu, Yu‐Bing Wang, Qiong Zhang, Ning‐Ning Song, Xi‐Yue Liu, Jia‐Yin Chen, Hong‐Wen Zhu, Bing‐Yao Zhou, Yun‐Chao Tao, Li Zhao, Ze‐Xuan Li, Yi‐Wei Li, Jia‐Qi Chen, Si‐Xin Tu, Cong‐Cong Qi, Sai‐Dan Ding, Gang Peng, Lin Xu, Yu‐Qiang Ding

**Affiliations:** ^1^ Shanghai Institute of Infectious Disease and Biosecurity Fudan University Shanghai China; ^2^ Laboratory Animal Center Fudan University Shanghai China; ^3^ Shanghai Yangzhi Rehabilitation Hospital (Shanghai Sunshine Rehabilitation Center) Tongji University School of Medicine Shanghai China; ^4^ Clinical Center for Brain and Spinal Cord Research Tongji University Shanghai China; ^5^ State Key Laboratory of Brain Function and Disorders and MOE Frontiers Center For Brain Science Institute of Brain Science Fudan University Shanghai China; ^6^ Precise Genome Engineering Center School of Life Sciences Guangzhou University Guangzhou China; ^7^ Key Laboratory of Animal Models and Human Disease Mechanisms Kunming Institute of Zoology the Chinese Academy of Science Kunming China; ^8^ Huashan Institute of Medicine (HS‐IOM) Huashan Hospital Fudan University Shanghai China

**Keywords:** excitatory and inhibitory balance, Gαs, inhibitory neuron, MINAR1, seizure susceptibility

## Abstract

Major Innate Disordered Notch2‐Associated Receptor 1 (MINAR1) is known to suppress angiogenesis and breast cancer cell growth and is associated with neurological disorders such as epilepsy. However, its neurobiological function remains unclear. Herein, we reveal the specific expression of MINAR1 in somatostatin (SST)‐ and parvalbumin (PV)‐positive interneurons in the mouse forebrain. To explore its functional significance, MINAR1 conditional knockout (CKO) mice were generated from Nestin‐Cre mice. During postnatal growth, gross brain morphology and cytoarchitecture were comparable between MINAR1 CKO mice and littermate controls; adult CKO mice exhibited increased vulnerability to pentylenetetrazole (PTZ)‐induced seizures, and this phenotype was also present in SST‐Cre–mediated CKO mice. Mechanistically, MINAR1 deficiency selectively impaired SST^+^ (but not PV^+^) interneuron excitability, reducing the inhibitory drive toward pyramidal neurons. This defect correlated with decreased G protein alpha S (Gαs) levels and disrupted Gαs–cAMP signaling. Notably, pharmacological activation of adenylate cyclase with forskolin rescued this inhibitory defect. Collectively, our results establish MINAR1 as a key regulator of seizure susceptibility, likely via Gαs–cAMP‐dependent modulation of SST^+^ interneurons, offering a molecular framework for developing targeted epilepsy therapies.

## Graphical abstract

Our study identifies MINAR1 as a novel regulator of cortical interneuron excitability and seizure susceptibility. MINAR1 is preferentially expressed in SST^+^ interneurons. Genetic ablation of MINAR1 leads to seizure hypersensitivity, reduced SST^+^ neuron excitability, and impaired Gαs–cAMP signaling, disrupting the E/I balance. These findings reveal a critical molecular mechanism underlying interneuronal dysfunction in epilepsy.

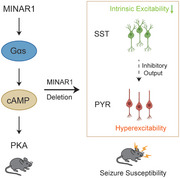

## 1 Introduction

Epilepsy is a neurological disorder that affects approximately 50 million people worldwide. It is characterized by recurrent seizures resulting from abnormal, excessive, or hypersynchronized neuronal activity in the brain [1, 2, 3, 4, 5, 6]. Accumulating evidence indicates that interneuron dysfunction plays a major role in epilepsy by disrupting the excitatory‐inhibitory (E/I) balance [7, 8, 9, 10, 11]. Gamma‐aminobutyric acid (GABA)ergic interneurons, which constitute approximately 20%–30% of neurons in the mammalian cerebral cortex, serve as key regulators of this balance [12, 13, 14, 15, 16]. Among these, parvalbumin‐positive (PV^+^) and somatostatin‐positive (SST^+^) neurons play particularly important roles by modulating cortical network activity through distinct inhibitory mechanisms [13, 17, 18]. Earlier studies have established that proper SST^+^ neuron function is essential for tuning endogenous network activity and preventing the switch to seizure‐like discharges under permissive conditions [19, 20, 21, 22, 23]. SST^+^ neurons influence neuronal response intensity to stimuli, thereby affecting perceptual sensitivity and the level of network synchrony [17, 24, 25, 26, 27, 28], whereas PV^+^ neurons contribute to rapid inhibition [9, 18, 29]. Given their critical roles, elucidating the molecular mechanisms underlying interneuronal dysfunction is essential for developing novel therapeutic approaches for epilepsy and related disorders.

Major Innate Disordered Notch2‐Associated Receptor 1 (MINAR1) was initially identified as a negative regulator of angiogenesis and growth through stabilization of Notch2 [6, 30, 31]. A subsequent study in zebrafish demonstrated that MINAR1 modulates hyperactivity by enhancing spinal cord neuronal activity and activating mTOR signaling [32]. Recent genetic studies have linked MINAR1 with neurobiological disorders. Whole‐exome sequencing analysis has identified a rare homozygous MINAR1 variant in patients with intellectual disabilities, psychomotor retardation, blindness, epilepsy, movement disorders, and cerebellar atrophy [33]. Additionally, missense mutations in MINAR1 were identified in three patients from the same family with intellectual disabilities, one of whom (the father) developed epilepsy at 40 years of age, while the other two patients (the children) remain under observation [34]. Given that MINAR1 is expressed in the mammalian brain (including in mice and humans) [30, 31, 32], further investigation of its neurobiological functions, particularly the consequences of its deficiency, is required to provide valuable insights.

Given its role in regulating neuronal activity and the overlapping neurological phenotypes observed in patients with MINAR1 variants, MINAR1 may influence key signaling pathways involved in neuronal excitability. One such pathway is the G protein‐coupled receptor (GPCR) signaling pathway, which plays a crucial role in modulating neuronal excitability and synaptic transmission [35, 36, 37, 38, 39, 40, 41]. Within this pathway, the G protein alpha S (Gαs), a stimulatory G protein, increases cyclic adenosine monophosphate (cAMP) levels by activating adenylyl cyclase. Elevated cAMP subsequently activates protein kinase A (PKA), which influences downstream signaling cascades. In neurons, Gαs primarily regulates intrinsic excitability via the cAMP–PKA axis [42, 43], and dysregulation of Gαs‐mediated signaling has been implicated in multiple neurological disorders, such as epilepsy, autism, and schizophrenia [44, 45, 46, 47, 48, 49, 50, 51]. However, despite its significance, the potential association between MINAR1 and GPCR signaling in disease pathogenesis has not yet been investigated.

In this study, we demonstrate that genetic deletion of MINAR1 in the mouse brain significantly increased the susceptibility to pentylenetetrazole (PTZ)‐induced seizures. Mechanistically, we found that MINAR1 deficiency reduces SST^+^ interneuron excitability, possibly by suppressing the Gαs–cAMP pathway, consequently impairing their inhibitory control over cortical pyramidal (PYR) neurons. Thus, our findings reveal for the first time that MINAR1 plays a critical role in mammalian brain function by regulating the activity of inhibitory interneurons, thereby modulating seizure susceptibility.

## 2 Materials and Methods

### 2.1 Animals

MINAR1‐flox mice were generated using CRISPR‐Cas9 technology to flank exon 2 of MINAR1 with loxP sites. The targeting construct included an enhanced green fluorescent protein (EGFP) reporter gene downstream of the MINAR1 coding sequence, separated by a P2A self‐cleaving peptide, to facilitate monitoring of expression. The targeting vector was electroporated into C57BL/6J mouse embryonic stem cells, and successful recombination events were verified via polymerase chain reaction (PCR) and Southern blot analysis. Chimeric mice obtained via blastocyst injection were bred with C57BL/6J mice to establish germline transmission. For MINAR1 deletion, MINAR1^flox/flox^ mice were crossed with Nestin‐Cre (selective deletion in the brain), VGAT‐Cre (GABAergic neuron–specific deletion), or SST‐Cre (SST^+^ interneuron–specific deletion) lines to generate the corresponding conditional knockout (CKO) models.

All mice were maintained on a C57BL/6J genetic background and housed in a specific pathogen‐free facility under a 12‐h light/dark cycle, with controlled temperature (22 ± 2°C) and humidity (50 ± 10%). Food and water were provided ad libitum.

Both male and female mice were equally represented in all behavioral, biochemical, and electrophysiological assays, and comparable outcomes were observed between the sexes (Figure S1); consequently, sex was not treated as a biological variable in our analyses.

### 2.2 Ethics Approval

All animal experiments were conducted in accordance with institutional guidelines and were approved by the Animal Care and Use Committee of the Laboratory Animal Center at Fudan University (permit DSF‐2020‐041). The study did not include human subjects or human‐derived samples.

### 2.3 Quantitative Real‐Time PCR (RT‐qPCR)

Total RNA was extracted from brain tissue using RNAiso Plus (9109; TaKaRa Biotechnology, Japan) according to the manufacturer's instructions and reverse transcribed to cDNA using PrimeScript RT Master Mix (RR037A; TaKaRa Biotechnology). RT‐qPCR was performed on an ABI‐Q7 real‐time PCR system (Applied Biosystems, CA, USA) using RT^2^ SYBR Green ROX qPCR Master Mix (330524; Qiagen, Germany). The primers used were as follows: MINAR1 forward (F): 5'‐CCGACAGTTTGTGAGCCTCT‐3'; MINAR1 reverse (R): 5'‐CTTACAGTCCATCTTCCGGCA‐3'; GAPDH forward (F): 5'‐GGAAGTGTTACGGTTTCAA‐3'; and GAPDH reverse (R): 5'‐ACAAGGATGGGGGTTACACA‐3'. Relative gene expression levels were calculated using the 2^−ΔΔCt^ method, with GAPDH used as an internal control [52].

### 2.4 Tissue Preparation

Mice were deeply anesthetized via intraperitoneal injection of sodium pentobarbital (40 mg/kg body weight) and transcardially perfused with 4% paraformaldehyde (PFA) in 1× phosphate‐buffered saline (PBS). After perfusion, the brains were carefully dissected, post‐fixed in 4% PFA at 4°C overnight, and cryoprotected in 20% sucrose/PBS until saturation. For cryosectioning, the brains were embedded in Tissue‐Tek O.C.T. compound (Sakura, Japan) and coronally sectioned at the desired thicknesses using a cryostat (RWD Life Science, China).

### 2.5 Nissl Staining

Brain sections were stained with 0.1% crystal violet (190‐M; Sigma‐Aldrich, MO, USA), dehydrated using a graded ethanol series, and covered with neutral balsam. Images were captured using an upright microscope (Ni‐E; Nikon, Japan).

### 2.6 Immunofluorescence Staining

Brain sections were subjected to antigen retrieval in 10 mm trisodium citrate and 2.5 mm citric acid at 95°C for 10 min. The sections were then blocked in 1% fetal bovine serum and 0.5% Triton X‐100 for 1 h at room temperature. Primary antibodies diluted in working solution were added and incubated at room temperature for 1 h, followed by overnight incubation at 4°C. The following day, after washing with PBS, the sections were incubated with secondary antibodies for 2 h at room temperature. For biotin‐conjugated secondary antibodies, an additional incubation (2 h at room temperature) was performed. Finally, the sections were covered with 75% glycerol in PBS and imaged using an upright (Ni‐E; Nikon) or confocal (FV3000; Olympus, Japan) microscope.

The primary antibodies used were as follows: rabbit anti‐CUX1 (1:300; 11733‐1‐AP; Proteintech, IL, USA), guinea pig anti‐CTIP2 (1:200; OB‐PGP012; Oasis), mouse anti‐TLE4 (1:200; sc‐365406; Santa Cruz Biotechnology, TX, USA), mouse anti‐SATB2 (1:300; sc‐81376; Santa Cruz Biotechnology), mouse anti‐PV (1:500; 235; Swant AG, Switzerland), guinea pig anti‐SST (1:500; OB‐PG9067; Oasis), and rabbit anti‐EGFP (1:500; OB‐PRB014; Oasis). The secondary antibodies used were as follows (diluted 1:500): donkey anti‐guinea pig‐488 (Oasis, GP488), donkey anti‐guinea pig‐Cy3 (706‐165‐148; Jackson ImmunoResearch, PA, USA), donkey anti‐guinea pig‐Cy5 (706‐175‐148; Jackson ImmunoResearch), donkey anti‐mouse‐488 (715‐545‐150; Jackson ImmunoResearch), donkey anti‐mouse‐Cy3 (715‐165‐150; Jackson ImmunoResearch), donkey anti‐mouse‐Cy5 (715‐175‐150; Jackson ImmunoResearch), goat anti‐guinea pig‐405 (106‐475‐003; Jackson ImmunoResearch), horse anti‐mouse‐biotinylated (BA‐2000‐1.5; Vector Labs, CA, USA), horse anti‐rabbit‐594 (DI‐1094‐1.5; Vector Labs), horse anti‐rabbit‐biotinylated (BA‐1100‐1.5; Vector Labs), streptavidin‐488 (SA‐5488‐1; Vector Labs), and streptavidin‐CY3 (SA‐1300‐1; Vector Labs).

### 2.7 Fluorescent In Situ Hybridization (FISH)

FISH was performed using the PinpoRNA 2.0 single‐color fluorescence detection system (PIF1000; Pinpoease, China) with a custom‐designed MINAR1 mRNA probe (Pinpoease), following the manufacturer's instructions. The sections were then subjected to immunofluorescence co‐staining with specific antibodies. Images were obtained using a confocal microscope (Olympus).

### 2.8 In Situ Hybridization (ISH)

Conventional ISH was performed as previously described [53, 54]. Riboprobes were designed based on protocols available from the Allen Brain Atlas website (http://www.brain‐map.org). DNA fragments corresponding to SST and PV were amplified from mouse brain cDNA. RNA probes were generated via in vitro transcription using digoxigenin‐labeled dUTP (DIG‐dUTP). The sections were treated with proteinase K for 30 min, post‐fixed in 4% PFA, and acetylated with acetic anhydride. DIG‐labeled probes were diluted in hybridization solution, and the slides were incubated at 63°C overnight. After washing, the sections were incubated with an anti‐DIG antibody (1:1000; alkaline phosphatase conjugate, sheep anti‐DIG, 11093274910; Roche, Switzerland) at 4°C overnight. The signals were visualized using an alkaline phosphatase substrate, and images were acquired using a Nikon upright microscope. To confirm virus‐mediated labeling of SST^+^ neurons, the proteinase K concentration was reduced from 10 µg/mL to 2 µg/mL during tissue pre‐treatment in the in situ hybridization procedure. Following visualization of PV mRNA, the sections were incubated with rabbit anti‐mCherry antibody (1:500; OB‐PRB013; Oasis) and developed using DAB to visualize virus‐infected cells. Images were acquired using a PanoPanel slide‐scanning microscope (Meca Scientific, China).

SST^+^ and PV^+^ neurons were quantified in coronal sections at approximately Bregma +0.5 mm. Neuronal density was calculated as the total number of neurons in the motor, somatosensory, and cingulate cortices divided by the combined area.

### 2.9 Electrode Implantation and Local Field Potential (LFP) Recordings

Mice were anesthetized with isoflurane (induction concentration of 3% and maintenance concentration of 1.5%) and then securely positioned in a stereotaxic frame (71000; RWD Life Science, China). A craniotomy was performed above the primary motor cortex (AP: +0.62 mm; ML: +1.5 mm). Subsequently, a custom‐made 16‐channel tungsten wire array electrode (35 µm diameter) was inserted to a depth of 0.7 mm below the dura mater and stabilized using dental cement. LFP recordings were obtained 1 week post‐implantation using an in vivo multichannel acquisition system (Apollo II; Eager Bio‐Tech, China). After establishing a baseline recording, PTZ (40 mg/kg) was administered intraperitoneally, and the mice were placed in a shielded, soundproof chamber for simultaneous video and LFP recordings over a period of 30 min.

LFP signals were amplified by a front‐end amplifier, band‐pass filtered between 0.1 Hz and 300 Hz, and saved as .plx files. MATLAB scripts were used for data analysis. Power spectral density (PSD) was calculated using the Welch method, applying a 5‐s Hamming window with 50% overlap. Frequency bands of the LFP signals were categorized as follows: δ (0.5–4 Hz), θ (4–8 Hz), α (8–13 Hz), β (13–30 Hz), low γ (30–60 Hz), and high γ (60–100 Hz). Interictal spikes were defined as transient events exceeding the baseline mean by 5 standard deviations. Epileptiform discharges were defined as high‐amplitude rhythmic activity lasting longer than 1 s.

### 2.10 PTZ‐Induced Seizure

PTZ (P6500; Sigma‐Aldrich) was dissolved in sterile saline to final concentrations of 3 and 4 mg/mL. Adult CKO mice and littermate controls received intraperitoneal PTZ injections at doses of 30 or 40 mg/kg, following established protocols [55, 56]. Immediately after injection, the mice were placed in a shielded, soundproof chamber, and their seizure behavior was video‐recorded for 30 min. Seizure severity was assessed using the Racine scale: 0, no response; 1, ear and facial twitching; 2, myoclonic jerks; 3, forelimb clonus; 4, generalized clonic seizures with rearing; and 5, generalized tonic‐clonic seizures with loss of posture. Behavioral scoring was performed by researchers blinded to mouse genotype.

### 2.11 Viral Vectors and Stereotaxic Injections

Adeno‐associated virus (AAV) vectors were used for cell type–specific labeling and optogenetic manipulation. The vectors used included AAV2/9‐PV vectors.Promoter.S5E2‐mCherry, AAV2/9‐emSST‐mCherry, AAV2/9‐mCaMKIIa‐EGFP, AAV2/9‐PV.Promoter.S5E2‐hChR2(H134R)‐mCherry, and AAV2/9‐emSST‐hChR2(H134R)‐mCherry (Taitool Bioscience, China). PV.Promoter.S5E2 is a promoter element that specifically targets cortical PV^+^ neurons [57], emSST is a promoter element that targets cortical SST^+^ neurons, and mCaMKIIa is a promoter element that targets cortical excitatory neurons. hChR2(H134R) is a human channel rhodopsin element containing an H134R point mutation. hChR2 opens inward cation channels to activate target neurons when illuminated with blue light (∼470 nm). The viral vectors were diluted to a final titer of 2 × 10^12^ viral genomes (V.G.)/mL.

For stereotaxic injections, 8‐week‐old mice were anesthetized with isoflurane and positioned in a stereotaxic frame. A small craniotomy was performed to expose the brain, and 50 nL of the virus was injected into the primary motor cortex at a rate of 2 nL/s using a microinjection pump. The injection needle remained in place for 5 min to allow proper viral diffusion before withdrawal. Electrophysiological experiments were conducted 1–2 weeks post‐injection to ensure adequate viral expression.

Viral labeling specificity refers to the accuracy of viral labeling of target cells and was defined as the percentage of cells that were co‐labeled with the virus and the immunolabeling of target cells out of the total number of cells labeled by the virus.

### 2.12 Patch‐Clamp Recordings

Patch‐clamp recordings were performed as previously described [58]. Briefly, mice were deeply anesthetized with sodium pentobarbital and transcardially perfused with an ice‐cold N‐methyl‐D‐glucamine (NMDG)‐based slicing solution containing (in mm): 110 NMDG, 2.5 KCl, 1.25 NaH_2_PO_4_, 25 NaHCO_3_, 25 glucose, 10 MgSO_4_, and 0.5 CaCl_2_ (pH 7.3–7.4; 300–310 mOsm). Brains were rapidly removed, and 300 µm‐thick coronal slices containing the primary motor cortex were prepared using a vibratome (VT1200S; Leica, Germany) in the same ice‐cold solution. The slices were first incubated in NMDG solution at 32°C for 10 min for recovery and then transferred to artificial cerebrospinal fluid (ACSF) containing (in mm): 120 NaCl, 2.5 KCl, 1.25 NaH_2_PO_4_, 26 NaHCO_3_, 10 glucose, 2 MgSO_4_, and 2 CaCl_2_ (pH 7.3–7.4, 300–310 mOsm) at room temperature for at least 1 h before recording. All solutions were continuously bubbled with 95% O_2_ and 5% CO_2_.

Whole‐cell patch‐clamp recordings were obtained using an upright microscope (Nikon) equipped with differential interference contrast optics and fluorescence imaging capabilities. Neurons expressing EGFP, mCherry, or both were visualized using appropriate filter sets. Recording pipettes (4–8 MΩ) were filled with either a potassium‐based internal solution (in mM: 135 K‐gluconate, 7 KCl, 10 HEPES, 0.5 EGTA, 4 Mg‐ATP, 0.4 Na‐GTP, and 10 Na‐phosphocreatine; pH 7.25; 290–300 mOsm) for current‐clamp recordings or a cesium‐based internal solution (in mM: 100 CsMeSO_3_, 60 CsCl, 10 HEPES, 0.2 EGTA, 4 Mg‐ATP, 0.3 Na‐GTP, and 5 QX‐314; pH 7.25; 290–300 mOsm) for recording light‐evoked and spontaneous inhibitory postsynaptic currents (light‐eIPSCs and sIPSCs, respectively).

For intrinsic excitability measurements, neurons were held at −70 mV in current‐clamp mode, and a series of depolarizing current steps (1000 ms duration, −100 pA to 500 pA in 20 pA increments) were applied to assess firing properties. The resting membrane potential was recorded immediately after establishing whole‐cell access. For action potential threshold recordings, a 1000 ms ramp current injection (−100 to 500 pA) was used to determine the spike threshold, defined as the membrane potential at which dV/dt first exceeded 10 V/s.

For optogenetic stimulation and recording, blue light pulses (473 nm, 2 ms, 0.1–5 mW/mm^2^ intensity) were generated by a laser (ThinkerTech, China) and delivered through an optical fiber controlled by a Digidata 1550B digitizer (Axon, USA). PYR neurons voltage‐clamped at −70 mV were recorded for both eIPSCs and sIPSCs in the presence of glutamatergic receptor blockers (CNQX, 20 µm; D‐AP5, 50 µm). For forskolin experiments, 20 µm of the compound (HY‐15371; MCE, China) was bath‐applied via circulating ACSF.

Electrophysiological signals were recorded using a MultiClamp 700 B amplifier (Axon) and digitized at 10 kHz (Digidata 1550B; Axon) using the pClamp 11 software (Molecular Devices, CA, USA). Signals were low‐pass filtered at a frequency of 2 kHz. Throughout recordings, series resistance (typically 10–20 MΩ) was continuously monitored, and data were excluded if resistance varied by > 20%. Data were analyzed offline using a custom MATLAB script.

### 2.13 Cell Culture and Mass Spectrometry (MS) Analysis

A MINAR1‐targeting guide RNA (5'‐CACCGCCAACCTTCTTGGACCATTG‐3') was cloned into the lentiCRISPR v2 vector. Lentiviral particles were generated in HEK293T cells, according to standard protocols. SH‐SY5Y cells were transduced and selected with puromycin (2 µg/mL) for 7 d to establish a stable MINAR1‐knockdown (KD) cell line.

MG132 was dissolved in dimethyl sulfoxide (DMSO) to prepare a 2 mm stock solution. When SH‐SY5Y cells reached 70% confluence, the stock solution was diluted 1:1000 in culture medium (final concentration, 2 µm); vehicle controls received DMSO at the same ratio. PYR41 was prepared in a similar manner using a 10 mm stock to achieve a final concentration of 10 µm.

For co‐immunoprecipitation assays, HEK293T cells were transfected with Flag‐tagged MINAR1 and/or HA‐tagged Gαs plasmids, either individually or in combination.

MS experiments were performed based on established protocols [59]. Proteins were extracted from control and MINAR1‐KD SH‐SY5Y cells using sodium dodecyl sulfate (SDS) lysis buffer (1% SDS, 10 mm TCEP, 40 mM CAA, and 100 mM Tris‐HCl, pH8.5). Proteins were extracted via sonication, denatured, and alkylated at 95°C for 10 min, followed by fluorometric quantification. Samples were digested with trypsin and desalted using a C18 StageTip. For liquid chromatography‐MS/MS analysis, 1 µg of peptides was analyzed using a Vanquish Neo UHPLC system coupled to an Orbitrap Exploris 480 mass spectrometer (Thermo Fisher Scientific, MA, USA). Peptides were separated on a 75 µm × 250 mm C18 column using a 60 min gradient. The MS parameters included a spray voltage of 2,400 V, an ion transfer tube temperature of 320°C, and data‐independent acquisition mode. Data were searched against the Swiss‐Prot database using Spectronaut v17 in spectral library‐free mode. Differential expression analysis was performed using the “limma” R package, and gene ontology enrichment and network analyses were conducted using “clusterProfiler” and Metascape.

### 2.14 Western Blot Analysis

Total protein was extracted from the cultured cells using RIPA buffer containing protease and phosphatase inhibitors. The samples were mixed with a one‐quarter volume of 5 × Loading Buffer (LT101L; Epizyme Biotech, MA, USA) and boiled at 100°C for 5 min. Equal amounts of protein were separated via 10% SDS polyacrylamide gel electrophoresis, and the separated proteins were transferred onto nitrocellulose membranes (HATF85R; Millipore, MA, USA). Membranes were blocked with 5% non‐fat milk in Tris‐buffered saline with Tween 20 (TBST) at room temperature for 1 h and incubated with primary antibodies at 4°C overnight. After washing with TBST, the membranes were incubated with horseradish peroxidase‐conjugated secondary antibodies (1:3000; Jackson ImmunoResearch) at room temperature for 1 h. Protein bands were visualized using an enhanced chemiluminescence reagent (32106; Thermo Fisher Scientific) and detected with a chemiluminescence imaging system (Tanon, China). Band intensities were quantified using ImageJ software.

### 2.15 Enzyme‐Linked Immunosorbent (ELISA)

The concentration of cAMP in cells was detected using an ELISA kit (D770001, Sangon Biotech, China), with the method fully following the instructions provided in the kit. In brief, after the cell culture was completed, 0.1 m HCl was added to dissolve the cells to obtain the test samples. Components and samples were added to the ELISA plate according to the instructions. Finally, the readings were taken using a microplate reader (Tristar 5, Berthold Technologies, Germany). The results were fitted to a third‐order polynomial linear regression curve of binding rate versus Log (cAMP standard concentration) using OriginPro 2024 software. The concentration of cAMP in the samples was then calculated based on the binding rate of the samples.

### 2.16 Statistical Analysis

All data are expressed as the mean ± standard error of the mean (SEM). Statistical analyses were performed using OriginPro 2024 software. Comparisons between two groups were performed using a two‐tailed independent Student's *t*‐test. Prior to the *t*‐test, normality was assessed using the Shapiro‐Wilk test, and homogeneity of variances was evaluated using Levene's test. Welch's correction was applied when variances were unequal. The magnitude of the observed differences was reported using Cohen's d as a measure of the effect size.

Comparisons among multiple groups were performed using one‐way ANOVA. Homogeneity of variances was evaluated using Levene's test. In cases of significant main effects, pairwise comparisons were performed using Fisher's Least Significant Difference (LSD) post‐hoc test. Two‐way repeated measures ANOVA was performed for two‐way factorial comparisons. Mauchly's test was used to assess sphericity of the repeated‐measures factor, and the Greenhouse‐Geisser correction was applied when the assumption was violated. Bonferroni correction was used for post‐hoc multiple comparisons.

Corrected significance levels are indicated in the relevant figure legends or in the Results section. For all tests, statistical significance was set at *p* < 0.05.

## 3 Results

### 3.1 Generation of MINAR1^Nestin^ CKO Mice

**FIGURE 1:**
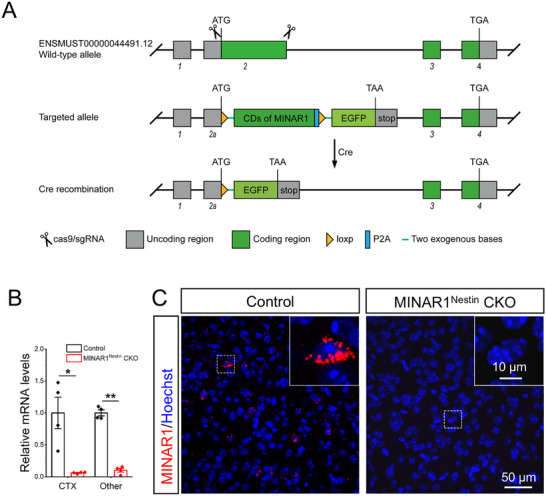
Construction of MINAR1^Nestin^ CKO mice using CRISPR‐Cas9 technology. (A) Schematic diagram illustrating the generation of MINAR1^flox/flox^ mice using CRISPR‐Cas9 technology. (B) Quantification of MINAR1 mRNA levels in the adult cerebral cortex and other brain regions of MINAR1^Nestin^ CKO and control mice by RT‐qPCR. Data are presented as means ± SEM. N = 4 mice per group. Student's *t* test, t_CTX_ = 3.770, ^*^ *P*_CTX_ < 0.05, Cohen's d_CTX_ = 2.704; t_Other_ = 16.29, ^**^ *P*_Other_ < 0.01, Cohen's d_Other_ = 11.52. “Other” denotes brain regions outside the cortex. (C) Detection of MINAR1 mRNA (red) in the cerebral cortex of adult MINAR1^Nestin^ CKO and control mice via FISH.

Nestin‐Cre mice were crossed with MINAR1^flox/flox^ mice to generate MINAR1^Nestin^ CKO offspring (Nestin‐Cre:MINAR1^flox/flox^) (Figure 1A). To verify MINAR1 deletion, RT‐qPCR was performed to measure MINAR1 mRNA levels in both the cerebral cortex and remaining brain regions of MINAR1^Nestin^ CKO mice. MINAR1 mRNA levels were reduced to extremely low levels compared with those in controls (Figure 1B). Owing to the lack of a reliable MINAR1 antibody, FISH was used to confirm knockout efficiency. Consistent with the RT‐qPCR results, almost no signals were detected in the cortex of MINAR1^Nestin^ CKO mice relative to controls (Figure 1C).

### 3.2 Normal Gross and Cellular Architecture in the MINAR1^Nestin^ CKO Brain

Body weight measurements revealed normal development of MINAR1^Nestin^ CKO mice during the first postnatal month, with no significant differences compared with the controls. However, by the second postnatal month, body weight began to decline progressively and reached statistical significance by the third month (Figure S3A). Despite this late‐onset reduction in body weight, gross brain morphology, including brain size and overall structure, exhibited no detectable differences between MINAR1^Nestin^ CKO and control mice (Figure S3B). CKO mice exhibited normal laminar architecture, as shown by Nissl staining, and preserved cortical layer patterning, as indicated by the comparable expression of layer‐specific markers (CUX1, CTIP2, and TLE4) (Figure S3C–E). Together, these findings indicate that neuron‐specific MINAR1 deletion affects body weight regulation in adulthood while maintaining normal brain development and cytoarchitecture.

### 3.3 Cellular Localization of MINAR1 in the Cerebral Cortex

We examined the temporal expression profile of MINAR1 in the mouse cerebral cortex. RT‐qPCR data showed that the mRNA levels of MINAR1 gradually increased from embryonic day 12.5 to postnatal day 7 and subsequently declined, reaching a relatively stable level after P21 (Figure S2).

**FIGURE 2:**
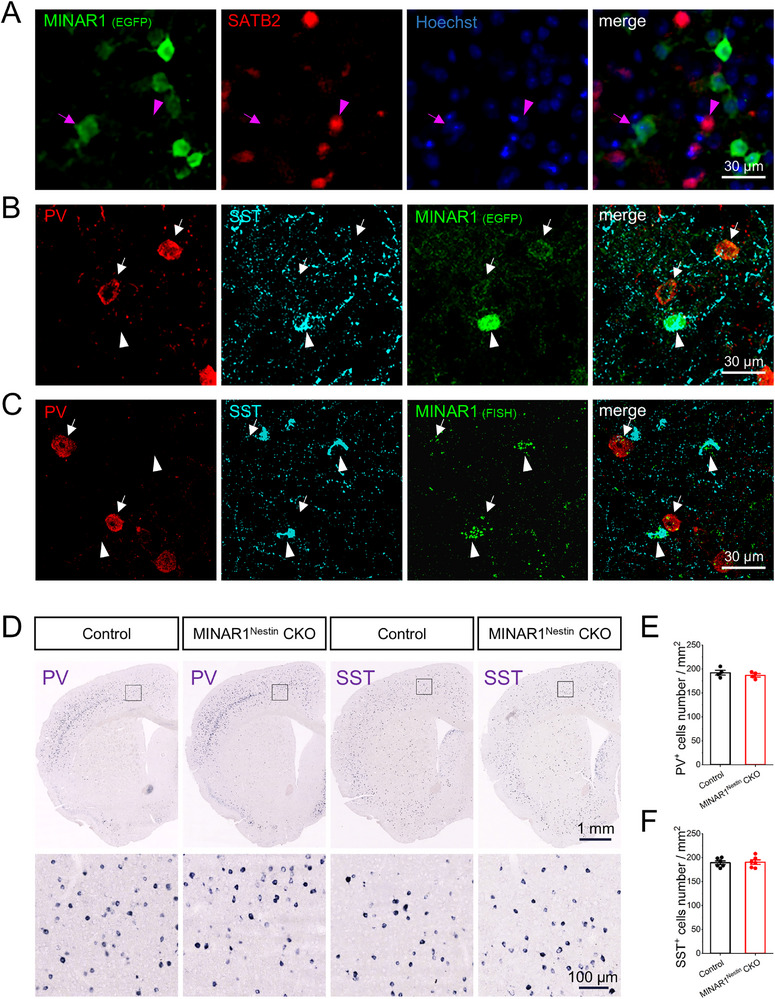
Cellular localization of MINAR1 in cortical interneurons and examination of PV^+^ and SST^+^ neurons in MINAR1^Nestin^ CKO mice. (A) Triple immunolabeling of Hoechst (blue), Satb2 (red), and EGFP (green) in the mouse cerebral cortex shows no co‐localization between EGFP‐labeled MINAR1^+^ neurons and Satb2‐labeled excitatory neurons. SATB2^+^ neurons (pink arrow) do not overlap with EGFP (MINAR1) neurons (pink arrow head). (B) Triple immunolabeling of PV (red), SST (cyan), and EGFP (green) reflecting MINAR1 protein levels in the cerebral cortex of adult MINAR1^flox/flox^ mice. PV^+^ neurons (arrowheads) exhibit low intensity of EGFP (MINAR1), whereas SST^+^ neurons (arrow) exhibit high intensity of EGFP (MINAR1). (C) Triple labeling of PV protein (red), SST protein (cyan) and MINAR1 mRNA (FISH; green) in the cerebral cortex of adult wild‐type mice. PV^+^ neurons (arrowhead) exhibit low levels of MINAR1 transcripts, whereas SST^+^ neurons (arrow) exhibit high levels of MINAR1 transcripts. (D) Distribution of PV and SST mRNA‐expressing neurons in the cortex of adult MINAR1^Nestin^ CKO and control mice. Boxed areas in the upper panels are magnified in the lower panels. (E,F) Quantification reveals no significant differences in the numbers of cortical PV^+^ and SST^+^ neurons between MINAR1^Nestin^ CKO and control mice.

To examine cellular distribution, we utilized MINAR1‐flox mice carrying an EGFP reporter inserted via a P2A peptide linkage to ensure proportional co‐expression of MINAR1 and EGFP. EGFP^+^ cells were scattered throughout the cortical layers, and no co‐localization of EGFP (MINAR1 reporter) and SATB2 (excitatory neuron marker) was observed in the cerebral cortex (Figure 2A), suggesting that MINAR1 is not expressed in cortical excitatory neurons.

Using EGFP, two distinct MINAR1^+^ neuronal populations were identified in the cerebral cortex: a high expression group (strong EGFP signal) and a low expression group (weak EGFP signal). Triple labeling for EGFP, PV, and SST showed weak EGFP signals in PV^+^ neurons and strong EGFP fluorescence in SST^+^ neurons in MINAR1^flox/flox^ mice (Figure 2B). FISH analysis further validated these findings by assessing MINAR1 expression via quantification of fluorescent dots. Triple labeling for MINAR1 (FISH, red), SST (immunostaining, cyan), and PV (immunostaining, green) showed similar results (Figure 2C), with 6.13 ± 0.50 MINAR1 mRNA puncta co‐localizing with PV neurons and 16.85 ± 0.94 with SST neurons. Among these, ∼30% corresponded to SST^+^ neurons (high expression), whereas ∼60% corresponded to PV^+^ neurons (low expression). These findings demonstrate a pronounced dichotomy in MINAR1 expression levels between major cortical inhibitory neuron subtypes, with SST^+^ neurons exhibiting approximately threefold higher expression than PV^+^ neurons.

Given previous reports implicating MINAR1 in cell development and differentiation [30], we investigated whether MINAR1 deficiency altered the density of cortical inhibitory neurons. Quantitative analysis revealed no statistically significant difference in the number of PV^+^ and SST^+^ neurons between adult control and CKO mice (Figure 2D–F).

### 3.4 Increased Seizure Susceptibility in MINAR1^Nestin^ CKO Mice

**FIGURE 3:**
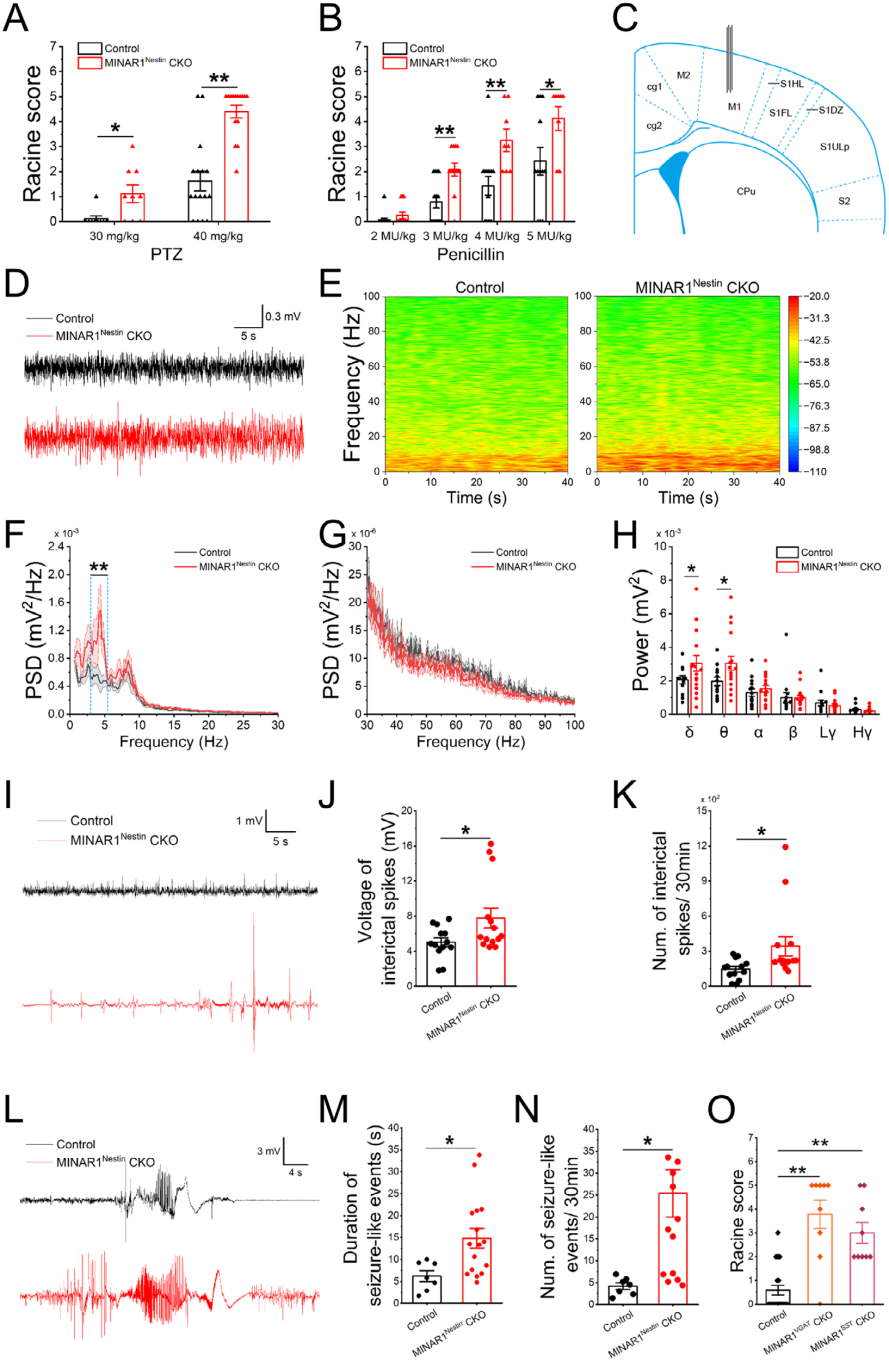
MINAR1^Nestin^ CKO mice exhibit increased severity of drug‐induced seizure behaviors with altered LFPs. (A) MINAR1^Nestin^ CKO mice exhibit increased seizure‐like behaviors compared with control mice after PTZ injection at doses of 30 and 40 mg/kg. No response was observed at a dose of 30 mg/kg in controls, whereas seizure‐like behaviors were observed in CKO mice. Data are presented as means ± SEM, N = 9 mice per group for 30 mg/kg, N = 15, 16 mice for 40 mg/kg (Student's *t*‐test, t_30 mg/kg_ = −2.714, ^*^ *P*_30 mg/kg_ = 0.01534, Cohen's d_30 mg/kg_ = −1.279; t_40 mg/kg_ = −5.803, ^**^ *P*_40 mg/kg_ < 0.01, Cohen's d_40 mg/kg_ = −2.085). (B) MINAR1^Nestin^ CKO mice exhibit increased seizure‐like behaviors compared with control mice after penicillin injection at doses of 3, 4 and 5 MU/kg. Data are presented as means ± SEM, N = 14, 12 mice for 2 MU/kg and 3 MU/kg, N = 14, 8 mice for 4 MU/kg and 5 MU/kg (Student's *t*‐test, t_2 MU/kg_ = −1.247, *P*_2 MU/kg_ = 0.2243, Cohen's d_2 MU/kg_ = −0.4907; t_3 MU/kg_ = −3.681, ^**^ *P*_3 MU/kg_ < 0.01, Cohen's d_3 MU/kg_ = −1.448; t_4 MU/kg_ = −3.024, ^**^ *P*_4 MU/kg_ < 0.01, Cohen's d_4 MU/kg_ = −1.340; t_5 MU/kg_ = −2.165, ^*^ *P*_5 MU/kg_ = 0.0440, Cohen's d_5 MU/kg_ = −0.9882). (C) Electrode implantation site in the primary motor cortex (M1) of adult mice. (D–H) MINAR1^Nestin^ CKO mice exhibit increased low‐frequency LFP activity induced by PTZ. Representative LFP traces (D). Time‐frequency energy analysis shows significantly increased energy in the low‐frequency band in CKO mice (E). PSD analysis of LFP in low‐frequency (F) and high‐frequency (G) bands. Specific rhythm energy intensity, showing significant increases in δ and θ rhythms in CKO mice (H), Student's *t* test, t_δ_ = −2.821, ^*^ *P*_δ_ = 0.01442, Cohen's d _δ_ = −1.880; t_θ_ = −2.466, ^*^ *P*_θ_ = 0.02832, Cohen's d_θ_ = −0.7547). (I) Representative traces of interictal spike discharges in a mouse following intraperitoneal PTZ injection. (J) MINAR1^Nestin^ CKO mice exhibit a significant increase in the amplitude of interictal spike discharges following intraperitoneal PTZ injection (Student's *t*‐test, t_Voltage_ = −2.159, ^*^ *P*_Voltage_ = 0.03978, Cohen's d_Voltage_ = −0.8316). (K) MINAR1^Nestin^ CKO mice exhibit a significant increase in the number of interictal spike discharges within 30 min following intraperitoneal PTZ injection (Student's *t*‐test, t_Num._ = −2.212, ^*^ *P*_Num._ = 0.0370, Cohen's d_Num._ = −0.8521). (L) Representative traces of epileptiform seizure discharges in MINAR1^Nestin^‐CKO and control mice following intraperitoneal PTZ injection. (M) MINAR1^Nestin^ CKO mice exhibit a significantly increased duration of epileptiform seizure discharges following intraperitoneal PTZ injection (Student's *t*‐test, t_Duration_ = −2.474, ^*^ *P*_Duration_ = 0.02772, Cohen's d_Duration_ = −1.121). (N) MINAR1^Nestin^ CKO mice exhibit a significantly higher number of epileptiform seizure discharges within 30 min post‐PTZ (Student's *t*‐test, t_Num._ = −2.644, ^*^ *P*_Num._ = 0.01525, Cohen's d_Num._ = −1.210). (O) Both MINAR1^VGAT^ CKO and MINAR1^SST^ CKO mice exhibit more severe seizure‐like behaviors compared with control mice following PTZ (40 mg/kg) injection (N = 22, 9, 9. Student's *t*‐test, t_VGAT_ = −6.475, ^**^ *P*_VGAT_ < 0.01, Cohen's d_VGAT_ = −2.215; t_SST_ = −5.680, ^**^ *P*_SST_ < 0.01, Cohen's d_SST_ = −2.110).

PV^+^ and SST^+^ interneurons are key inhibitory neurons that regulate the E/I balance in neural networks, a critical determinant of normal brain function and seizure modulation [9, 27]. To assess seizure susceptibility, we used PTZ, a GABA_A_ receptor antagonist commonly used to induce seizures in animal models. At a subthreshold dose (30 mg/kg), control mice exhibited no epileptiform behavior, whereas MINAR1^Nestin^ CKO mice exhibited clear seizure‐like activity (Figure 3A). Increasing the dose to 40 mg/kg induced tonic‐clonic seizures in MINAR1^Nestin^ CKO mice, whereas the controls exhibited markedly milder symptoms (Figure 3A). To further validate these findings, we administered penicillin, another seizure‐inducing agent that inhibits GABA release and competitively blocks GABA receptors [60]. At a subthreshold dose (2 MU/kg), neither group exhibited overt epileptic behavior (Figure 3B). In contrast, higher doses (3 and 5 MU/kg) triggered seizures in both groups, with MINAR1^Nestin^ CKO mice consistently showing more severe seizure phenotypes than the controls (Figure 3B). Notably, no spontaneous seizures were observed in MINAR1^Nestin^ CKO mice under baseline conditions.

To further evaluate seizure susceptibility, we recorded LFPs in the cortex after PTZ injection (40 mg/kg). During the interictal period, MINAR1^Nestin^ CKO mice exhibited significantly increased δ‐ and θ‐band power compared with controls (Figure 3C–H). In addition, quantitative analysis of interictal spikes revealed a marked increase in both spike frequency and amplitude in MINAR1^Nestin^ CKO mice (Figures 3I–K). Seizure‐like discharges were also more frequent and prolonged in mutants than in controls (Figure 3L–N). Notably, baseline LFP power spectra (δ, θ, α, β, and γ) did not differ between genotypes under resting conditions (Figure S4). Together, these findings suggest that MINAR1 deletion in inhibitory neurons predisposes mice to drug‐induced epilepsy, likely through impaired interneuron‐mediated inhibition.

### 3.5 Increased Seizure Susceptibility in MINAR1VGAT and MINAR1SST CKO Mice

The specific expression of MINAR1 in cortical inhibitory neurons suggests that PV^+^ interneurons, SST^+^ interneurons, or both may contribute to PTZ‐induced seizure susceptibility. However, because Nestin‐Cre drives pan‐neuronal MINAR1 deletion, the observed seizure phenotype in MINAR1^Nestin^ CKO mice cannot be exclusively attributed to inhibitory neuronal dysfunction. To resolve this issue, we generated CKO mice with selective MINAR1 deletions in the GABAergic neurons (VGAT‐Cre:MINAR1^flox/flox^) or SST^+^ interneurons (SST‐Cre:MINAR1^flox/flox^). Quantification of the number of cortical PV^+^ and SST^+^ neurons revealed no significant differences between adult MINAR1^VGAT^ and MINAR1^SST^ CKO mice (Figure S5). Following PTZ administration (40 mg/kg, i.p.), both MINAR1^VGAT^ and MINAR1^SST^ CKO mice exhibited more severe tonic‐clonic seizures than controls (Figure 3O). These results confirm that inhibitory neurons, particularly SST^+^ interneurons, play a central role in modulating PTZ‐induced seizure susceptibility.

### 3.6 Reduced Excitability of SST^+^ Neurons in MINAR1Nestin CKO Mice

**FIGURE 4:**
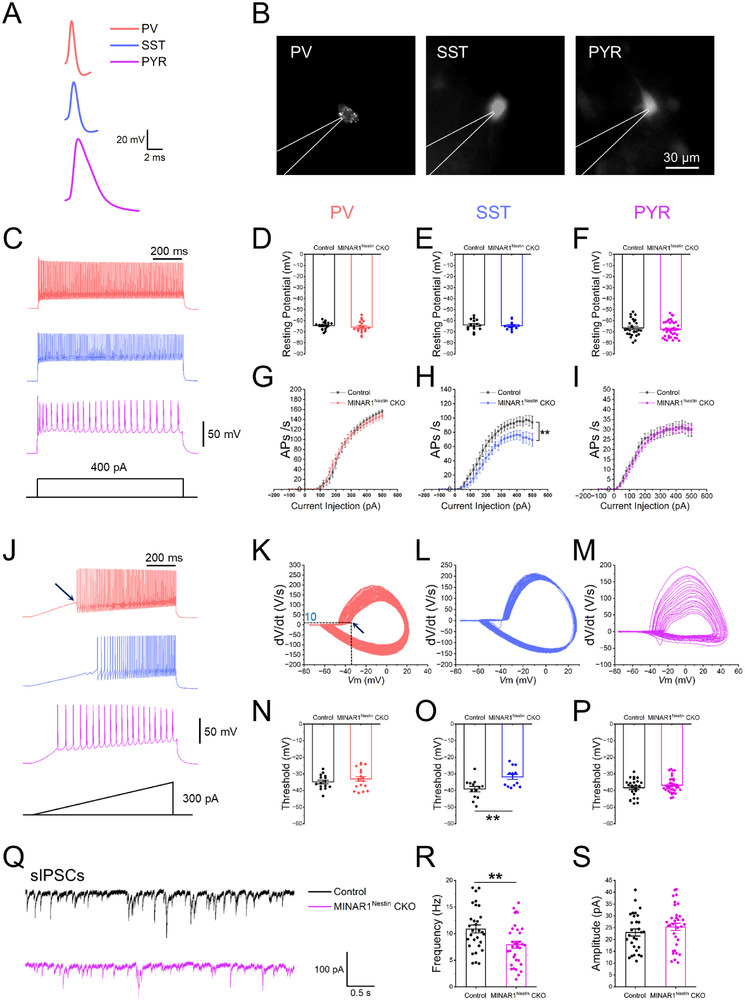
Reduced excitability of SST^+^, but not PV^+^ or pyramidal (PYR) neurons, in MINAR1^Nestin^ CKO mice. (A) Representative action potential recordings of PV^+^, SST^+^, and PYR neurons. (B) Images of PV^+^, SST^+^, and PYR neurons recorded in whole‐cell patch‐clamp mode. Neurons were labeled with AAV2/9‐PV.Promoter.S5E2‐mCherry, AAV2/9‐emSST‐mCherry, and AAV2/9‐mCaMKIIa‐EGFP viruses, respectively. Control group: PYR neurons from 9 mice (*n* = 27); PV^+^ neurons from 6 mice (*n* = 16); SST^+^ neurons from 6 mice (*n* = 15). MINAR1^Nestin^ CKO group: PYR neurons from 13 mice (*n* = 37); PV^+^ neurons from 5 mice (*n* = 19); SST^+^ neurons from 6 mice (*n* = 13). (C) Action potential responses of PV^+^, SST^+^, and PYR neurons to step current injections ranging from ‐100 to 500 pA (20 pA increments) at 400 pA. (D–F) Resting membrane potentials of the three types of neurons show no significant changes between the two genotypes. (G–I) Changes in action potential frequencies of the three types of neurons under step current stimulation. PYR and PV^+^ neurons show no significant changes, while SST^+^ neurons exhibit a significant decrease in action potential frequency in MINAR1^Nestin^ CKO mice compared with controls. Data are presented as means ± SEM. Two‐way repeated measures ANOVA: genotype effect: F[1, 24] = 2373, ^**^ *p* < 0.01, η^2^ = 0.990; current effect: F[30, 720] = 79.11, ^**^ *p* < 0.01, η^2^ = 0.767; interaction: F[30, 720] = 1.177, *p* = 0.3249, η^2^ = 0.047. (J) Action potentials of PV^+^, SST^+^, and PYR neurons under ramp current injections ranging from −100 pA to 500 pA. (K–M) Voltage derivative vs. voltage during ramp current stimulation of PV^+^, SST^+^, and PYR neurons. The dashed line in (K) indicates the voltage corresponding to the first action potential during the ramp current stimulation, at which dV/dt exceeds 10 V/s, representing the potential threshold. (N–P) Potential thresholds of PV^+^ (N) and PYR neurons (P) in MINAR1^Nestin^ CKO mice are unchanged, whereas those of SST^+^ neurons (O) exhibit a significant increase. (Student's *t*‐test, *t*_Threshold_ = −3.226, ^*^ *p*_Threshold_ < 0.01, Cohen's d_Threshold_ = −1.265). (Q) Representative traces of sIPSCs. (R,S) sIPSC frequency is significantly reduced (R), whereas amplitude is unchanged (S) in PYR neurons from MINAR1^Nestin^ CKO mice (neurons from seven mice in the control group, *n* = 27; neurons from six mice in the MINAR1*^Nestin^* CKO group, *n* = 34; Student's *t*‐test, t_Frequency_ = 2.980, ^*^ *P*_Frequency_ < 0.01, Cohen's d_Frequency_ = 0.7465).

To determine whether MINAR1 deletion alters inhibitory interneuron activity, we performed patch‐clamp recordings of the cortical neurons in MINAR1^Nestin^ CKO mice. We stereotaxically injected AAV2/9‐PV.Promoter.S5E2‐mCherry (labeling PV^+^ neurons), AAV2/9‐emSST‐mCherry (labeling SST^+^ neurons), and AAV2/9‐mCaMKIIa‐EGFP (labeling PYR neurons) viruses into the primary motor cortex. Co‐labeling of mCherry with PV or SST revealed high efficiency and specificity for the virus (Figure S6). Electrophysiological recordings were conducted 2 weeks post‐injection. Notably, PV^+^ and PYR neurons in MINAR1^Nestin^ CKO mice exhibited resting membrane potential, action potential properties, and excitation thresholds comparable to those observed in the controls (Figure 4A–D,F,G,I–K,M,N,P). In contrast, SST^+^ neurons exhibited a significantly reduced action potential firing frequency and an elevated excitation threshold in MINAR1^Nestin^ CKO mice, despite unaltered resting membrane potentials (Figure 4A–C,E,H,J,L,O). These findings demonstrate that MINAR1 deficiency selectively impairs the excitability of SST^+^ interneurons but not that of PV^+^ interneurons or PYR neurons in the cortex.

To investigate potential alterations in inhibitory synaptic transmission, we examined whether MINAR1 deletion affects the synaptic output from SST^+^ or PV^+^ interneurons to PYR neurons. We selectively expressed channelrhodopsin‐2 (ChR2) in PV^+^ or SST^+^ interneurons by injecting AAV2/9‐PV.Promoter.S5E2‐hChR2(H134R)‐mCherry or AAV2/9‐emSST‐hChR2(H134R)‐mCherry into the motor cortex, respectively. Whole‐cell patch‐clamp recordings were obtained from PYR neurons during optogenetic stimulation of transfected interneurons. Our results revealed no significant differences in the light‐eIPSCs of PYR neurons between MINAR1^Nestin^ CKO and control mice when either PV^+^ or SST^+^ interneurons were optically stimulated (Figure S7). This indicates that despite impaired intrinsic excitability of SST^+^ neurons (as shown previously), evoked synaptic transmission from both PV^+^ and SST^+^ interneurons to PYR neurons remains functionally preserved in MINAR1^Nestin^ CKO mice.

We next analyzed sIPSCs in PYR neurons. Using specific AAV vectors targeting PV^+^ and SST^+^ neurons, we investigated the contribution of these inter‐neuronal populations to sIPSCs in PYR neurons. Although the amplitude and frequency of PV^+^ neurons–mediated sIPSCs remained unchanged, a significant reduction in the frequency of SST^+^ neurons–mediated sIPSCs was observed in MINAR1^Nestin^ CKO mice compared with controls (Figure 4Q–S). These findings demonstrate that MINAR1 deficiency specifically impairs SST^+^ neuron‐mediated inhibition of PYR neurons. Importantly, this effect cannot be attributed to altered synaptic transmission function (Figures S7–S9) but instead likely reflects the reduced intrinsic excitability of SST^+^ neurons observed in our previous experiments (Figure 4).

### 3.7 Impaired Gαs‐Dependent cAMP Signaling in MINAR1 Deficient Cells

**FIGURE 5:**
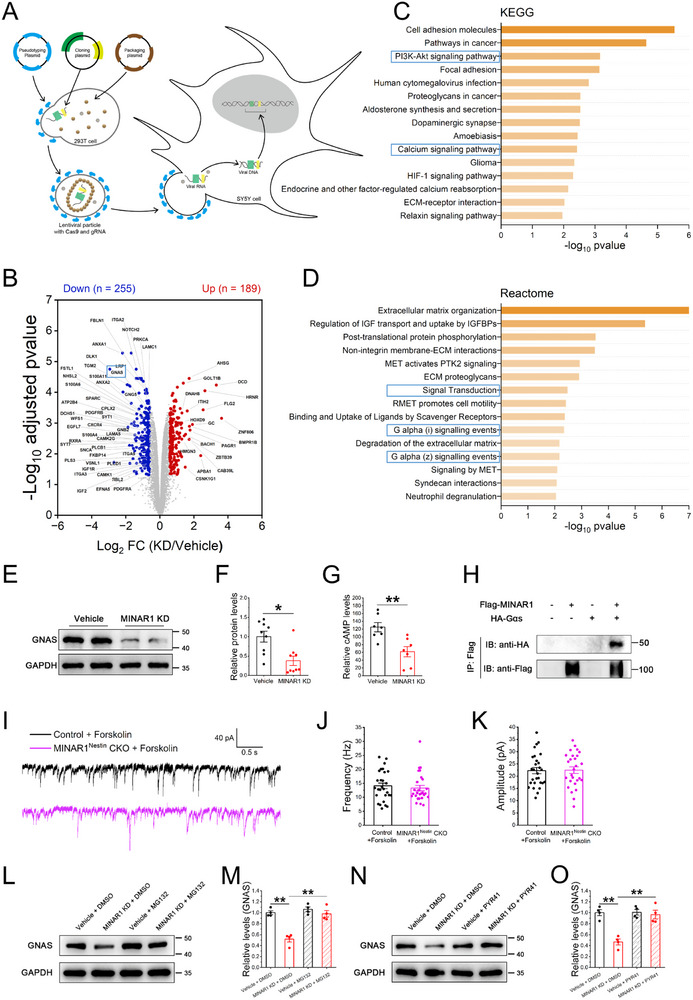
MINAR1 deficiency leads to impairment of Gαs‐dependent cAMP pathway. (A) Schematic diagram illustrating the construction of the MINAR1 KD SH‐SY5Y cell line. (B) MS‐based differential protein analysis in the cell lines, highlighting downregulation of GNAS (encoding the Gαs protein; blue box). (C,D) KEGG and Reactome pathway analyses of differentially expressed proteins highlight strong associations with key signaling pathways, including the PI3K–Akt pathway, calcium signaling, and Gαs protein–regulated pathways. (E,F) Immunoblotting analysis of total proteins reveals a substantial decrease in Gαs protein levels of MINAR1 KD SH‐SY5Y cells relative to the controls. Data are presented as means ± SEM, *N* = 9 wells of cells for each group (Student's *t*‐test, *t*_GNAS_ = 3.411, ^**^ *P*_GNAS_ < 0.01, Cohen's d_GNAS_ = 1.6088). (G) cAMP content is significantly decreased in MINAR1 KD cells (*N* = 7 wells for each group). Student's *t*‐test, t_cAMP_ = 3.539, ^**^ *P*_cAMP_ < 0.01, Cohen's d_cAMP_ = 1.891). (H) Representative traces of sIPSCs under Forskolin incubation. (I) Anti‐Flag immunoprecipitation from cells co‐expressing MINAR1‐Flag and Gαs‐HA show an HA‐reactive band absent, verifying MINAR1‐Gαs interaction. (J,K) Forskolin treatment restores the frequency of sIPSCs in cortical PYR neurons of MINAR1^Nestin^ CKO mice to levels comparable to those of control mice. (L,M) Reduced Gαs protein levels in MINAR1 KD cells are fully restored after incubation with MG132 (Student's *t*‐test, t_Gαs_ = −5.647, ^**^ *P*_Gαs_ < 0.01, Cohen's d_Gαs_ = −3.993). Each group was tested in triplicate using four wells per six‐well plate. (N,O) Reduced Gαs protein levels in MINAR1 KD cells are fully resorted after incubation with PYR41 (Student's *t‐*test, t_Gαs_ = −5.155, ^**^ *P*_Gαs_ < 0.01, Cohen's d_Gαs_ = −3.645). Each group was tested in triplicate using four wells per six‐well plate.

To elucidate the molecular mechanisms underlying reduced SST^+^ neuron excitability in MINAR1^Nestin^ CKO mice, we performed in vitro studies using a CRISPR‐Cas9‐generated MINAR1 KD SH‐SY5Y cell line (Figure 5A). Quantitative proteomic analysis of MINAR1‐deficient cells revealed significant alterations in MS‐based protein expression profiles (Figure 5B). Kyoto Encyclopedia of Genes and Genomes (KEGG) and Reactome pathway enrichment analyses identified three major signaling cascades affected by MINAR1 loss: PI3K–Akt signaling, calcium signaling pathways, and Gαs‐mediated signal transduction (Figure 5C,D). Notably, MS analysis revealed a significant reduction in Gαs (GNAS) protein levels, which was further confirmed by western blot analysis (Figure 5E,F). These findings suggest that MINAR1 deficiency disrupts Gαs‐dependent signaling, potentially through reduced Gαs protein levels, which may contribute to the reduced excitability of SST^+^ neurons.

As a key regulator of GPCR signaling, Gαs activates adenylyl cyclase to promote cAMP production. Consistent with the observed reduction in Gαs protein levels, enzyme‐linked immunosorbent assay measurements revealed significantly decreased cAMP concentrations in MINAR1‐deficient SH‐SY5Y cells (Figure 5G). To investigate the interaction between MINAR1 and Gαs, HEK293T cells were co‐transfected with MINAR1‐Flag and Gαs‐HA expression constructs. The cell lysates were subjected to immunoprecipitation using an anti‐FLAG antibody, followed by western blot analysis using an anti‐HA antibody. An HA‐positive signal was detected only in cells co‐expressing both constructs, indicating a direct physical interaction between MINAR1 and Gαs (Figure 5H). To establish the functional consequences of this pathway disruption, we pharmacologically rescued this deficit by applying forskolin (a direct activator of adenylyl cyclase) during sIPSC recordings in PYR neurons. Remarkably, forskolin treatment fully restored the frequency of SST^+^ neuron‐mediated sIPSCs in MINAR1^Nestin^ CKO mice to control levels (Figures 5I–K). Together, these findings demonstrate that MINAR1 deficiency reduces Gαs protein abundance, attenuates cAMP signaling, and reduces SST^+^ neuron excitability, thereby increasing susceptibility to PTZ‐induced seizures. The complete rescue by forskolin establishes the Gαs–cAMP pathway as the primary mechanism linking MINAR1 to interneuron dysfunction and seizure predisposition.

### 3.8 MINAR1 Stabilizes Gαs Protein by Preventing Ubiquitin‐Mediated Degradation

To elucidate how MINAR1 regulates Gαs protein stability, we investigated potential proteasomal degradation mechanisms. Treatment with the proteasome inhibitor MG132 (2 nm) [61] significantly restored Gαs protein levels in MINAR1 KD SH‐SY5Y cells, although levels remained slightly lower than those of controls (Figure 5L,M). This suggests that MINAR1 deficiency enhances proteasome‐dependent degradation of Gαs.

Building on previous work showing that MINAR1 reduces ubiquitination‐mediated degradation of DEPTOR [31], we next examined whether ubiquitination contributes to Gαs instability in MINAR1‐deficient cells. Treatment with PYR41 (10 µM), a specific inhibitor of ubiquitin‐activating enzyme E1, fully restored Gαs protein levels in MINAR1 KD cells to control values (Figure 5N,O). These results demonstrate that MINAR1 normally protects Gαs from ubiquitin‐proteasome degradation. In the absence of MINAR1, Gαs undergoes enhanced ubiquitination and subsequent proteasomal degradation. This post‐translational regulatory mechanism is crucial for maintaining normal Gαs protein levels and neuronal function. The complete rescue by PYR41 specifically implicates ubiquitination as the key degradation pathway responsible for reduced Gαs stability in MINAR1‐deficient cells.

## 4 Discussion

In this study, we identified a novel role for MINAR1 in regulating cortical interneuron excitability and seizure susceptibility. We showed that MINAR1 was preferentially expressed in cortical inhibitory neurons, with particularly higher levels in SST^+^ interneurons than in PV^+^ neurons (Figure 2B,C). Genetic ablation of MINAR1 in interneurons led to marked hypersensitivity to pharmacologically induced seizures (Figure 3), specifically reduced excitability of SST^+^ neurons (Figure 4H,O), and impairment of Gαs–cAMP–dependent signaling (Figure 5E–O), likely disrupting the E/I balance in local neural networks. Together, these findings establish MINAR1 as a critical regulator of cortical inhibition and reveal a novel molecular mechanism underlying inter‐neuronal dysfunction in epilepsy.

Our study revealed a previously unrecognized spatiotemporal specificity of MINAR1 expression in the forebrain. MINAR1 expression levels strongly correlated with the developmental stage, gradually increasing from embryonic day 12.5, peaking at postnatal day 7, and then progressively declining to a relatively stable baseline level in adulthood (Figure S2). However, no overt defects in brain architecture or changes in the number of cortical PV^+^ and SST^+^ neurons were observed in MINAR1^Nestin^ CKO mice (Figure 2; Figures S3 and S5), possibly due to the relatively high tolerance of the MINAR1 protein to variations [33]. Although previous studies have reported MINAR1 expression across various tissues, including the central nervous system [30, 31, 32], our data demonstrate for the first time its exclusive localization to cortical PV^+^ and SST^+^ interneurons, with particularly higher expression in SST^+^ neurons. PV^+^ and SST^+^ neurons are the two major interneuron populations that differ in morphology, electrophysiological properties, and functions [13, 62, 63, 64]. Notably, these subtypes differentially regulate PYR neuronal activity: SST^+^ neurons target distal dendrites to modulate input integration, whereas PV^+^ neurons innervate the soma and proximal dendrites to control output generation [65, 66, 67, 68, 69]. The preferential expression of MINAR1 in SST^+^ interneurons suggests its potential importance in dendritic inhibition, fine‐tuning of synaptic integration, and regulation of input‐output transformations in cortical networks. This cell type–specific expression pattern implies that MINAR1 plays distinct roles in different inhibitory circuits, with particularly critical functions in dendrite‐targeting SST^+^ interneurons.

Beyond its preferential expression in SST^+^ neurons, the heightened seizure susceptibility observed in MINAR1^Nestin^ CKO, MINAR1^VGAT^ CKO, and MINAR1^SST^ CKO mice provides compelling evidence that MINAR1 is essential for maintaining cortical E/I balance. Previous studies have also demonstrated that SST^+^ neurons play a critical role in regulating endogenous network activity and controlling the transition to seizure‐like discharges under specific conditions [19, 20]. Our findings indicate that dysfunction of SST^+^ interneurons is the primary driver of the epileptogenic phenotype observed in MINAR1 CKO mice. MINAR1 expression was threefold higher in SST^+^ neurons than in PV^+^ neurons (Figure 1). MINAR1 deletion resulted in a reduction of Gαs protein levels, likely through increased ubiquitin‐proteasome degradation (Figure 5H,L–O) and led to diminished Gαs activity, likely reducing basal adenylyl cyclase activity and cAMP concentrations in SST^+^ terminals (Figure 5E–G). cAMP‐PKA phosphorylation of Kv1.2 and HCN channels is necessary for high‐frequency burst firing characteristic of SST^+^ Martinotti cells [65, 70, 71, 72, 73], and the loss of MINAR1 may slow recovery from inactivation of these channels, thereby raising the current threshold for action potential initiation. SST^+^ terminals target the distal apical dendrites of layer‐V pyramidal neurons [65, 74]. When these terminals fail to fire, dendritic Ca^2+^ spikes, normally shunted by GABA‐B‐mediated inhibition, become larger and more frequent, thereby lowering the induction threshold for NMDA‐dependent burst firing.

Synapses on the soma of the SST^+^ and PV^+^ mice remained intact (Figure S8), and somatic inhibition was preserved. Consequently, the mutant cortex was still able to generate normal oscillations under normal conditions (Figure S4). However, in the presence of PTZ, the functions of GABA_A_ receptors may be blocked or attenuated, and the weakened dendritic inhibition is unmasked, allowing Ca^2+^ spikes to propagate to the soma and trigger prolonged depolarizing plateaus that manifest as interictal spikes in LFPs (Figure 3I–K). Bath application of forskolin, which bypasses Gαs and directly re‐elevates cAMP, restored SST^+^ firing (Figure 5I–K), suggesting that the Gαs–cAMP axis is critical for the PTZ‐induced seizure phenotype. Thus, we hypothesize that MINAR1 does not function as a diffuse “brake,” but rather stabilizes a precisely localized Gαs pool that maintains cAMP‐dependent dendritic inhibition. The loss of this molecular interaction is sufficient to shift the cortical E/I balance toward hypersynchrony and seizure generation.

However, our finding that loss of MINAR1 reduces the intrinsic excitability of SST^+^ interneurons should be interpreted with caution. All electrophysiological analyses in this study were performed in MINAR1^Nestin^ CKO mice but not in MINAR1^SST^ CKO animals. Moreover, there are currently no direct in vivo experimental data showing that MINAR1 regulates Gαs ubiquitination. Thus, future studies will be required to replicate these recordings in mice harboring MINAR1 deletion exclusively in SST^+^ neurons and to probe whether MINAR1 controls Gαs ubiquitination in vivo, thereby solidifying our conclusions.

It remains unclear why MINAR1 deletion specifically impairs the electrophysiological properties of SST^+^ neurons, characterized by reduced action potential firing frequency, elevated threshold potentials, and diminished inhibitory output to PYR neurons, while sparing PV^+^ neurons. We speculate that this cell type‐specific vulnerability arises from three converging factors. First, expression strength: MINAR1 mRNA density in PV^+^ cells is only 36% of that in SST^+^ cells (Figure 2B, C). Second, functional redundancy: PV^+^ neurons co‐express Gαolf (Gnal), which couples to adenylyl cyclase with an efficiency comparable to Gαs, whereas SST^+^ neurons lack detectable Gαolf expression [75, 76, 77]. Third, channel phenotype: rapid spiking in PV^+^ cells relies on Kv3.1/3.2 channels, whose gating properties exhibit minimal sensitivity to cAMP‐PKA phosphorylation [78, 79, 80], whereas SST^+^ cells depend on Kv1.2 and HCN channels, which are direct PKA substrates [65, 70, 71, 72, 73]. Because MINAR1 deletion reduced total Gαs levels by ∼60% (Figure 5F), the residual Gαs together with Gαolf may remain sufficient to sustain the modest cAMP levels required for PV^+^ firing but may fall below the normal threshold required to potentiate dendritic inhibition in SST^+^ cells. Thus, MINAR1 loss may selectively compromise SST^+^ interneuron function simply because these cells operate closer to the lower end of the Gαs–cAMP dynamic range. Future studies are needed to explore this idea.

The Gαs–cAMP signaling regulates neuronal excitability through multiple mechanisms [35, 81], including modulation of voltage‐gated ion channel function via phosphorylation [36, 82, 83, 84], regulation of synaptic plasticity and gene expression, and control of action potential dynamics, among others. Although our study establishes the role of MINAR1 in this pathway, several important questions remain unresolved, including the identification of specific ion channel targets, potential cell type‐specific effects across different neuronal populations, and the temporal dynamics of cAMP signaling disruption. Nevertheless, these findings position MINAR1 as a novel regulator of fundamental excitability mechanisms through its maintenance of Gαs–cAMP signaling fidelity. The precise molecular targets downstream of this pathway represent an important direction for future research.

The functional relationship between MINAR1 and Gαs–cAMP signaling poses several important mechanistic questions. Our study suggests that MINAR1, via its interaction with Gαs, stabilizes Gαs protein levels and functions by reducing Gαs ubiquitination and its subsequent degradation. However, key aspects of this regulation, particularly its spatial organization and the temporal dynamics of Gαs stabilization, remain unresolved. Future investigations should employ proximity ligation assays to map interaction domains as well as live‐cell imaging to study real‐time dynamics.

Our findings contribute to a better understanding of E/I imbalance disorders by identifying MINAR1 as a novel regulator of interneuron function, revealing that diminished SST^+^ cell intrinsic excitability weakens dendritic inhibition and providing potential therapeutic targets for epilepsy, autism spectrum disorders, and schizophrenia [12, 85, 86, 87]. These diseases share a common pathophysiology involving GABAergic interneuron impairment, particularly an E/I imbalance. Interestingly, the MINAR1–Gαs–cAMP axis represents a promising new pathway for therapeutic intervention in these disorders.

## Funding

This research was supported by the STI 2030‐Major Projects (2022ZD0204900), National Natural Science Foundation of China (32271072), Natural Science Foundation of Shanghai (22ZR1466300, 23ZR1413500), Collaborative Innovation Program of the Shanghai Municipal Health Commission (2020CXJQ01), Shanghai Municipal Science and Technology Major Project (2018SHZDZX01), and Zhangjiang Laboratory.

## Author Contributions

**Wei – Tang Liu**: conceptualization, data curation, writing the original draft, formal analysis, visualization, project administration, and investigation. **Ning – Ning Song**: supervision.**Cong – Cong Qi**: supervision. **Sai – Dan Ding**: supervision. **Gang Peng**: writing – review & editing. **Lin Xu**: conceptualization, methodology, writing – review & editing. **Yu – Qiang Ding**: conceptualization, methodology, resources, supervision, writing, review, and editing. **Zhi – Bin Hu**: investigation. **Ling Hu**: investigation. **Yu – Bing Wang**: investigation. **Qiong Zhang**: investigation. **Xi – Yue Liu**: investigation. **Jia – Yin Chen**: investigation. **Hong – Wen Zhu**: investigation. **Bing – Yao Zhou**: investigation. **Yun – Chao Tao**: investigation. **Li Zhao**: investigation. **Ze – Xuan Li**: investigation. **Yi – Wei Li**: investigation. **Jia – Qi Chen**: investigation. **Si – Xin Tu**: investigation.

## Conflicts of Interest

The authors declare no conflict of interest.

## Supporting information




**Supporting File**: advs74261‐sup‐0001‐SuppMat.docx.

## Data Availability

The data that support the findings of this study are available from the corresponding author upon reasonable request.
