# Supplementary material for: Cortical Somatostatin Neurons Regulate Seizure Susceptibility via MINAR1/Gαs–cAMP Signaling
Source: Adv Sci (Weinh). 2026 Feb 4;13(21):e19388. doi: 10.1002/advs.202519388 (PMC13073241; doi:10.1002/advs.202519388)
Supplement: Supplementary file 1 — Supporting File: advs74261‐sup‐0001‐SuppMat.docx. [file ADVS-13-e19388-s001.docx]

Supporting Information


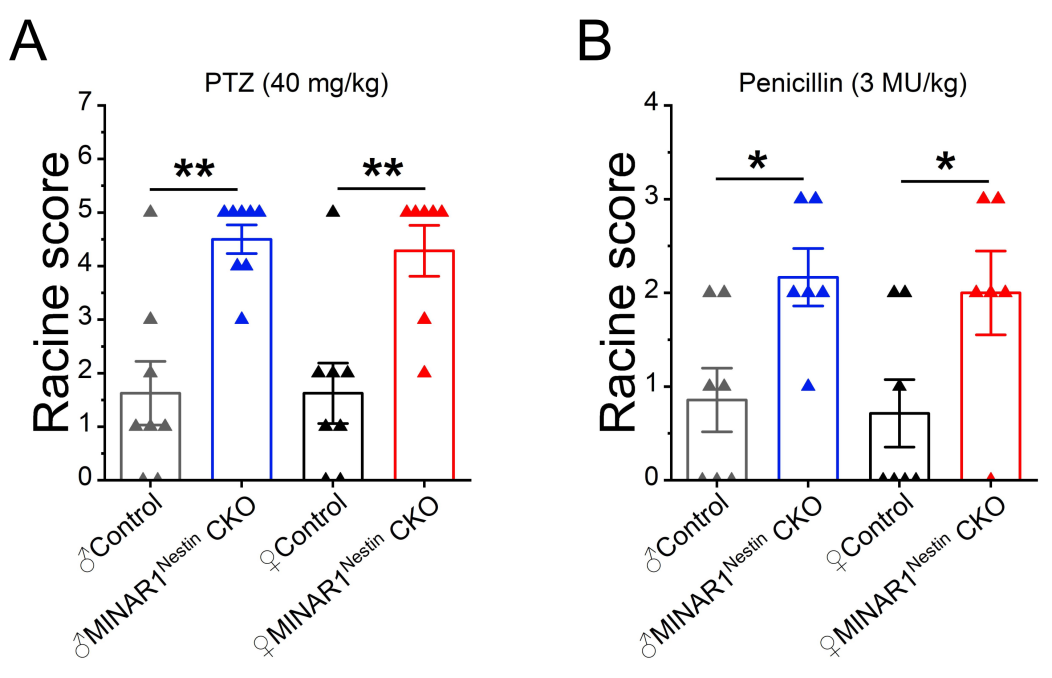


**Supplementary Fig. S1. MINAR1^Nestin^ CKO mice exhibit an increased severity of drug-induced seizure behaviors with no gender difference.**

**A.** Both male and female MINAR1^Nestin^ CKO mice exhibit more severe seizure-like behaviors than controls after 40 mg/kg PTZ injection, with no difference between sexes. Data are means ± SEM, One-way ANOVA, ♂Control vs ♂MINAR1^Nestin^ CKO: CI [-4.287, -1.463], ***P* < 0.01, Cohen's d = -2.202; ♀Control vs ♀MINAR1^Nestin^ CKO: CI [-4.123, -1.199], ***P* < 0.01, Cohen's d = -1.836; ♂MINAR1^Nestin^ CKO vs ♀MINAR1^Nestin^ CKO: CI [-1.248, 1.676], *P* = 0.7659, Cohen's d = 0.211. N = 8, 8, 8, 7 mice.

**B.** Both male and female MINAR1^Nestin^ CKO mice exhibit more severe seizure-like behaviors than controls after 3 MU/kg penicillin injection, without gender difference. Data are means ± SEM, One-way ANOVA, ♂Control vs ♂MINAR1^Nestin^ CKO: CI [-2.385, -0.234], **P* < 0.05, Cohen's d = -1.566; ♀Control vs ♀MINAR1^Nestin^ CKO: CI [-0.950, 1.283], ***P* < 0.01, Cohen's d = -1.261; ♂MINAR1^Nestin^ CKO vs ♀MINAR1^Nestin^ CKO: CI [-2.361, -0.210], **P* < 0.05, Cohen's d = 0.177. N = 7, 6, 7, 6 mice.


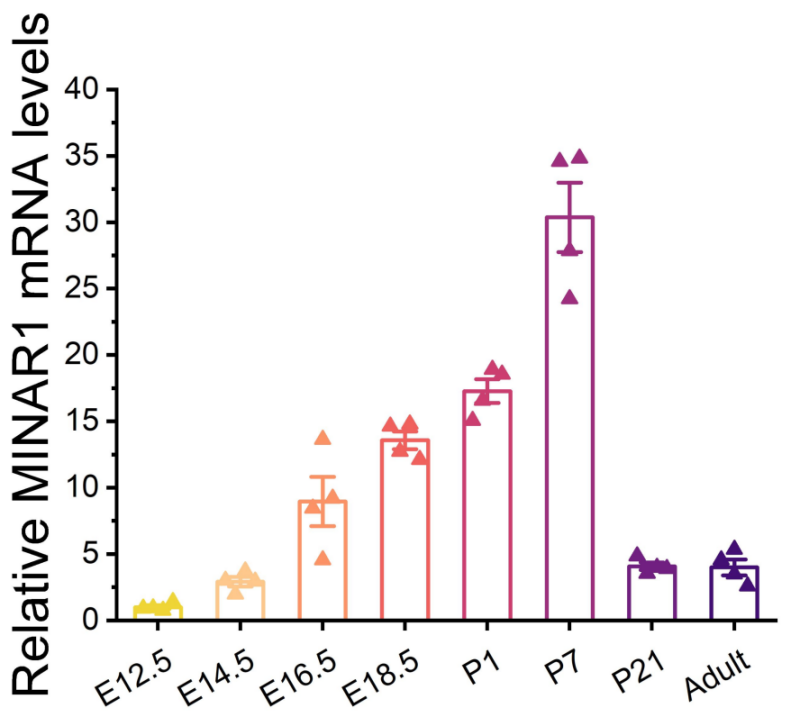


**Supplementary Fig. S2. Temporal expression profile of Minar1 in the mouse cerebral cortex.**

RT-qPCR was used to quantify MINAR1 mRNA levels in the mouse cortex from embryonic days E12.5, E14.5, E16.5, E18.5 through postnatal days P1, P7, P21 to adulthood (N = 4 per time point).


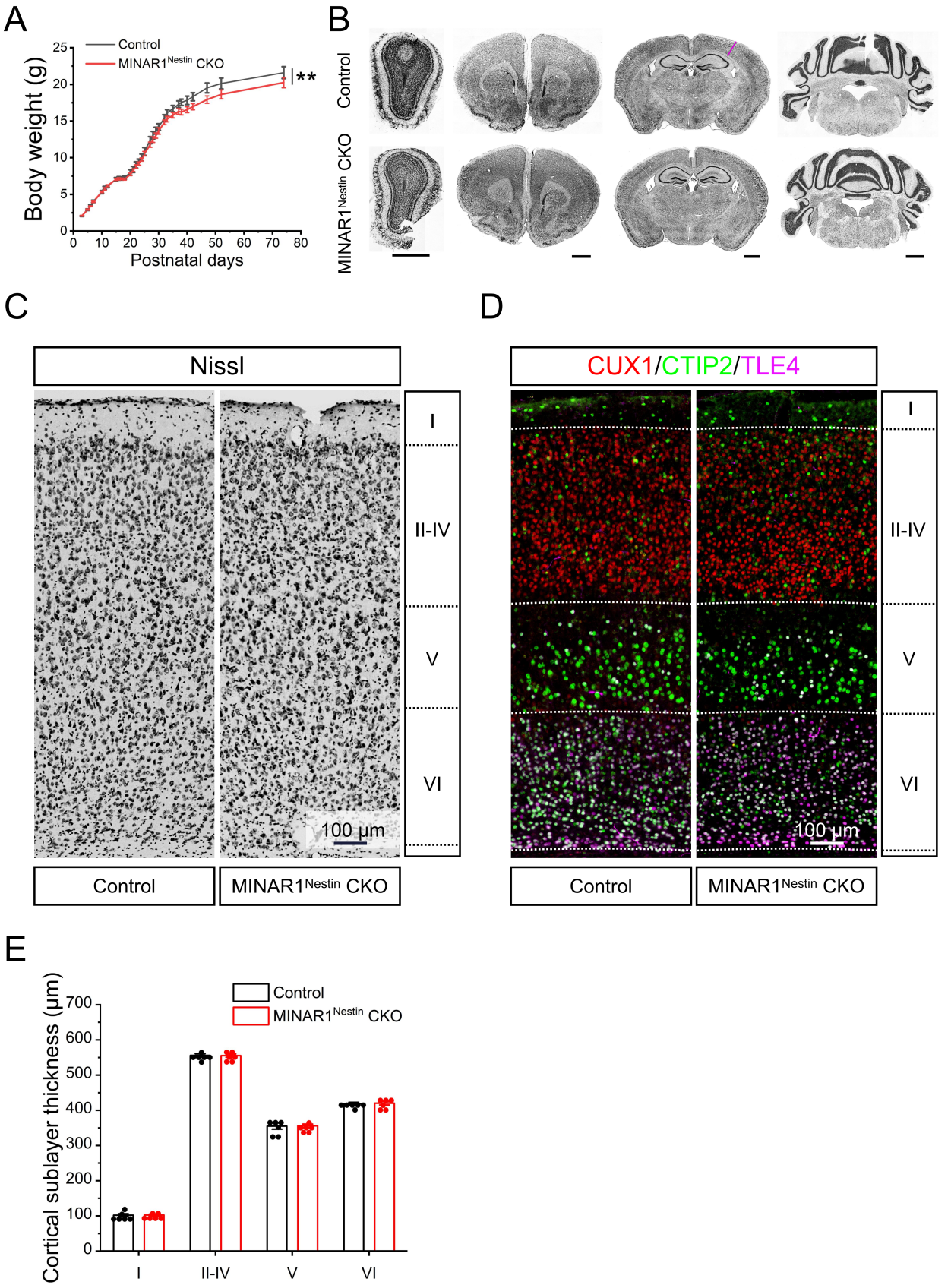


**Supplementary Fig. S3. Brain architecture is not altered in MINAR1^Nestin^ CKO mice.**

**A.** The growth curve of MINAR1^Nestin^ CKO mice showing a reduction of body weight in adulthood. Data are means ± SEM. Two-way repeated measures ANOVA: genotyping effect: F[1, 14] = 864.8, ***P* < 0.01, η^2^ = 0.984; time effect: F[30, 420] = 398.5, ***P* < 0.01, η^2^ = 0.966; interaction: F[30, 420] = 0.9422, *P* = 0.4017, η^2^ = 0.063, N = 8 mice per group.

**B.** Nissl staining shows no detectable alterations in the olfactory bulb, forebrain, thalamus, hypothalamus and brain stem of MINAR1^Nestin^ CKO mice compared with controls. Scale bar = 1 mm.

**C, D.** Representative images of Nissl staining (C) and immunofluorescence (D) shows no obvious changes in the cerebral cortex of MINAR1^Nestin^ CKO mice relative to controls. CUX1, CTIP2 and TLE4 are genes expressed in layers II-IV, V-VI and V, respectively.

**E.** No significant changes in cortical sublayer thickness were observed in MINAR1^Nestin^ CKO mice (measured in the S1FL cortex at Bregma +0.98 mm on coronal sections; N = 6 mice for each group).


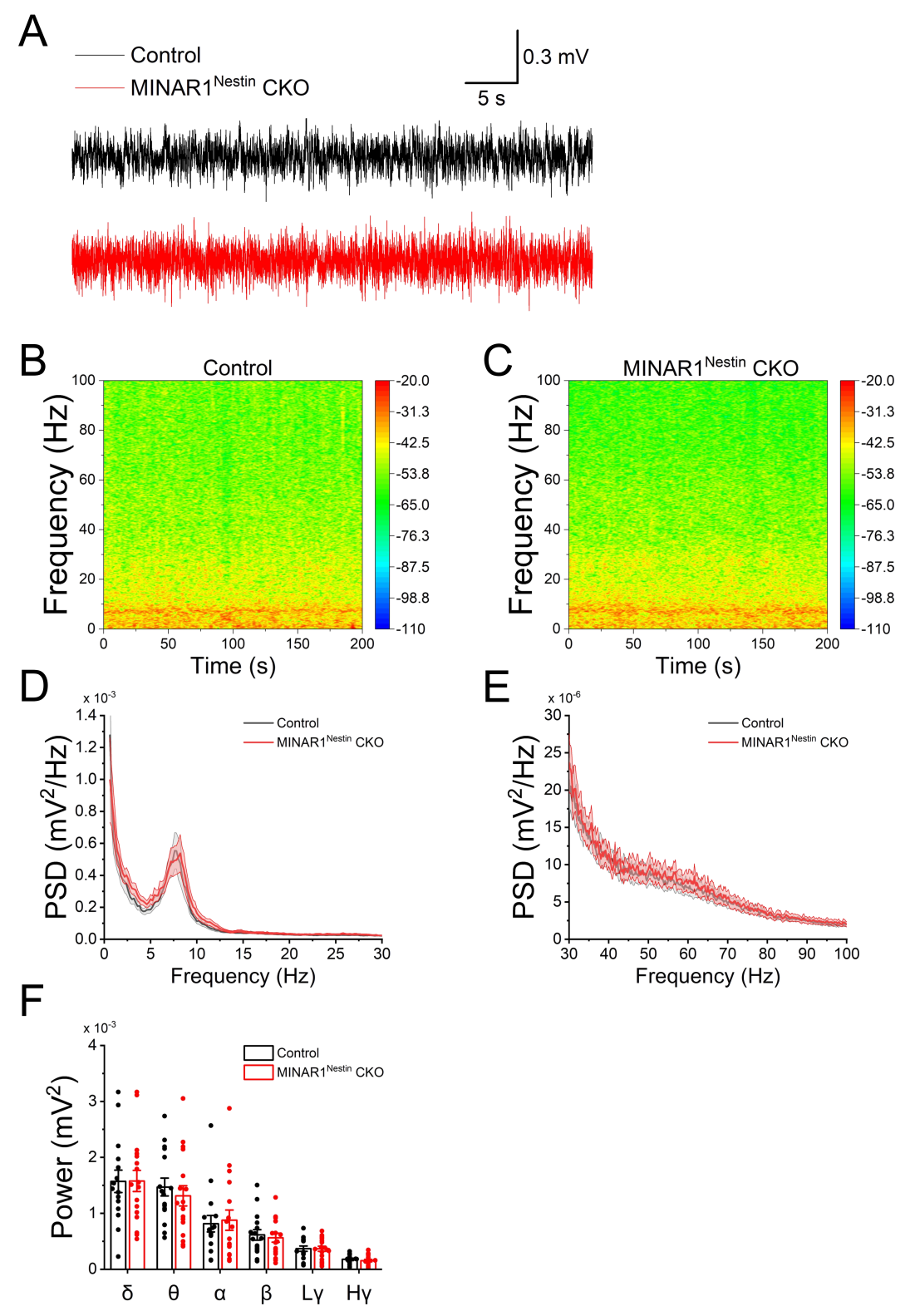


**Supplementary Fig. S4. No significant differences in cortical field potentials are observed between MINAR1^Nestin^ CKO and control mice.**

**A.** Representative traces of cortical LFP in MINAR1^Nestin^ CKO and control mice.

**B, C.** No obvious differences in time-frequency energy analysis for both groups of mice.

**D, E.** PSD analysis of LFP in the low-frequency and high-frequency bands shows no significant differences.

**F.** No significant differences in the energy intensity of various rhythms of cortical field potentials are found between MINAR1^Nestin^ CKO and control mice. Data are means ± SEM, N = 16 mice for each group.


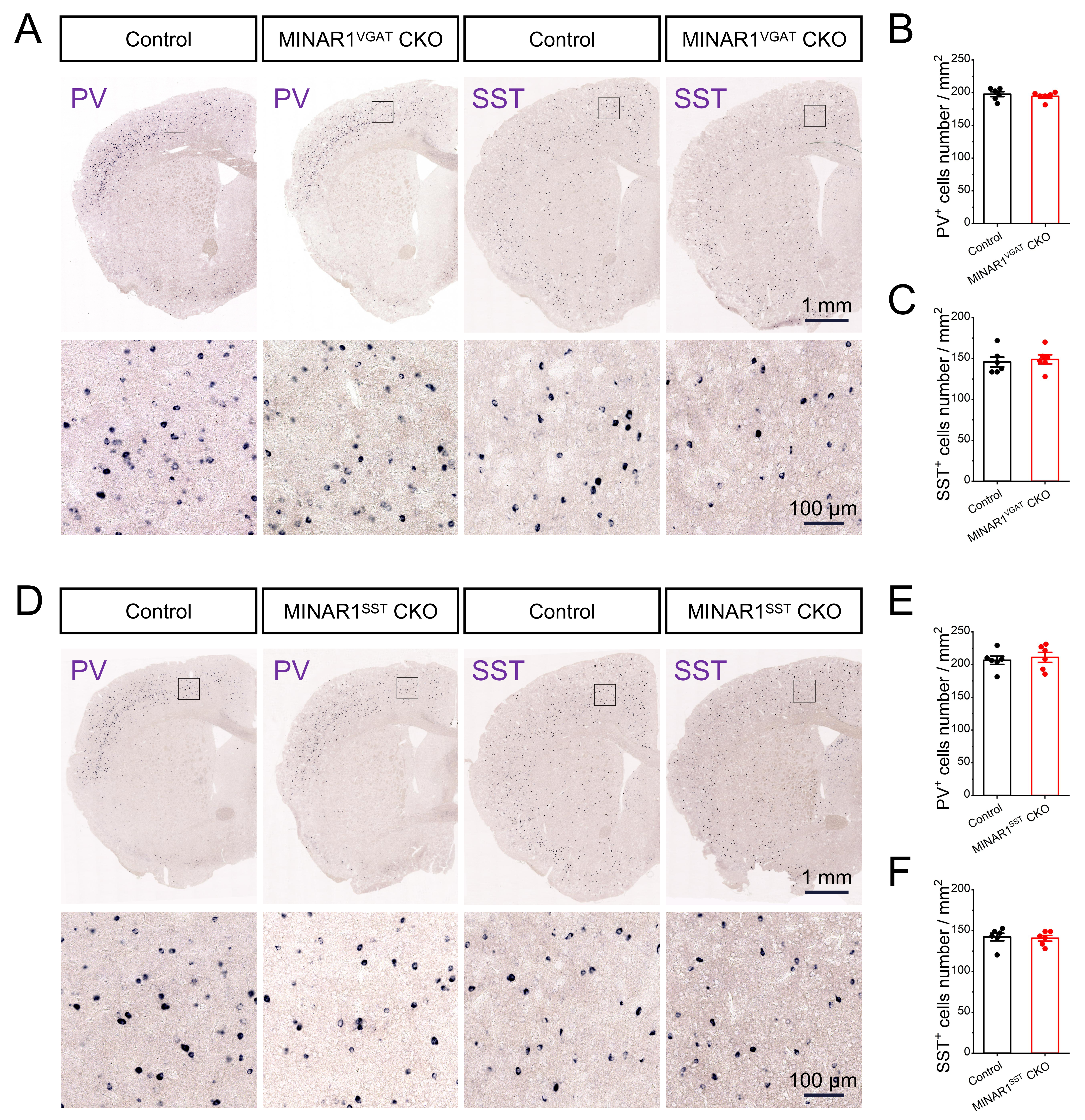


**Supplementary Fig. S5. The number of cortical PV^+^ and SST^+^ neurons remained unchanged in the MINAR1^VGAT^ and MINAR1^SST^ CKO mice.**

**A.** The distribution of neurons containing PV and SST mRNA in the cortex of adult MINAR1^VGAT^ CKO and control mice. Boxed areas in upper panels are enlarged in lower panels.

**B, C.** No significant differences in the numbers of PV^+^ and SST^+^ neurons between the MINAR1^VGAT^ CKO mouse cortex. N = 6 mice for each group.

**D.** The distribution of neurons containing PV and SST mRNA in the cortex of adult MINAR1^VGAT^ CKO and control mice. Boxed areas in upper panels are enlarged in lower panels.

**E, F.** The number of cortical PV^+^ and SST^+^ neurons shows no significant differences in the MINAR1^SST^ CKO ,mice compared with controls. N = 6 mice for each group.


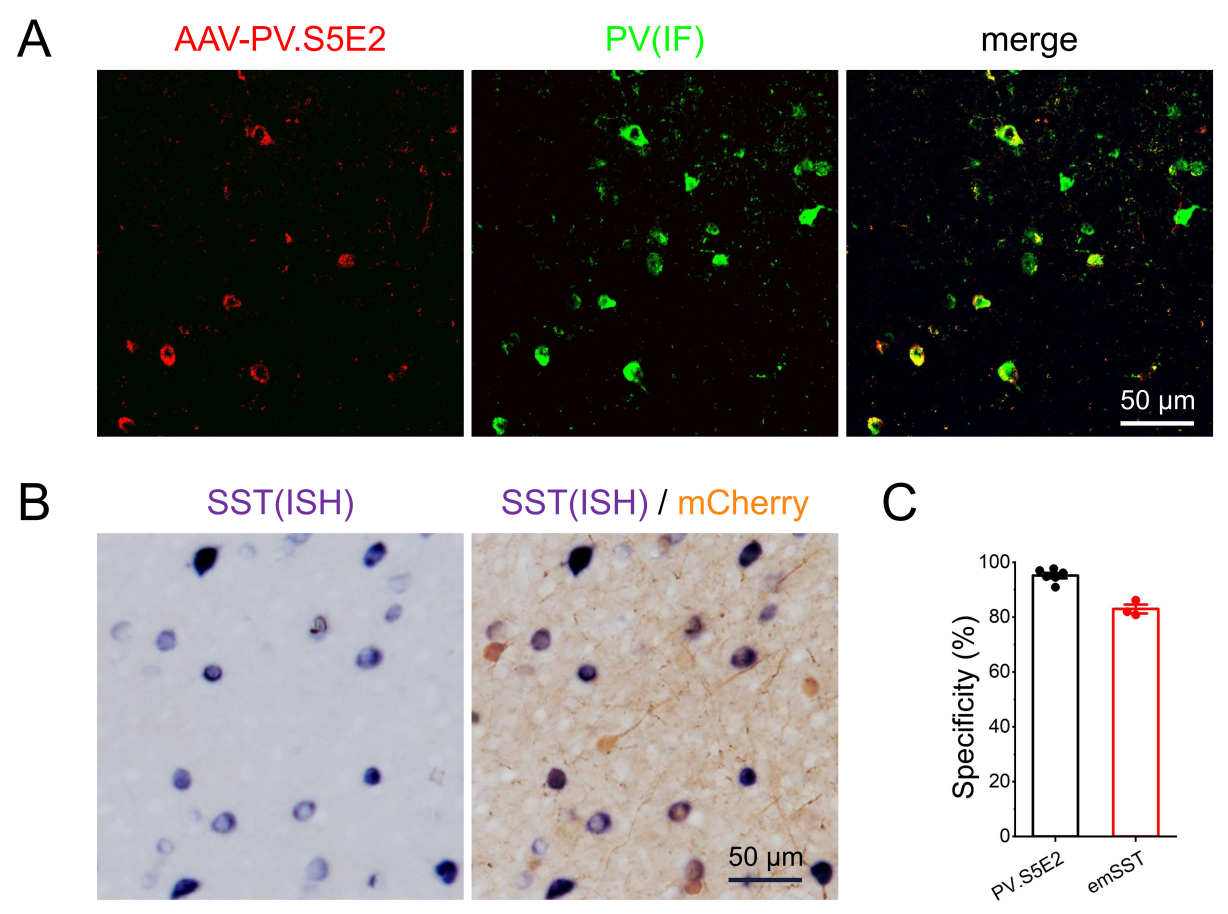


**Supplementary Fig. S6. Validation of specificity for labeling PV^+^ neurons with the S5E2 promoter and SST^+^ neurons with the emSST promoter in the mouse cortex.**

**A.** Representative image showing co-labeling of virus-labeled cells (red) and PV immunofluorescence staining (green) in the cortex 8 days after the injection of AAV2/9-PV.Promoter.S5E2-mCherry.

**B.** Representative image from the AAV2/9-emSST-mCherry injection site showing co-stainting of SST^+^ cells labeled by *in situ* hybridization of SST mRNA (blue-purple) and virus-labeled cells with mCherry immunohistochemistry (reddish-brown). Eight days post-injection.

**C.** PV^+^ neurons labeled with the S5E2 promoter and SST^+^ neurons labeled with the emSST promoter both show high targeting accuracy in the mouse cortex. The two groups contained 6 and 3 mice, respectively.


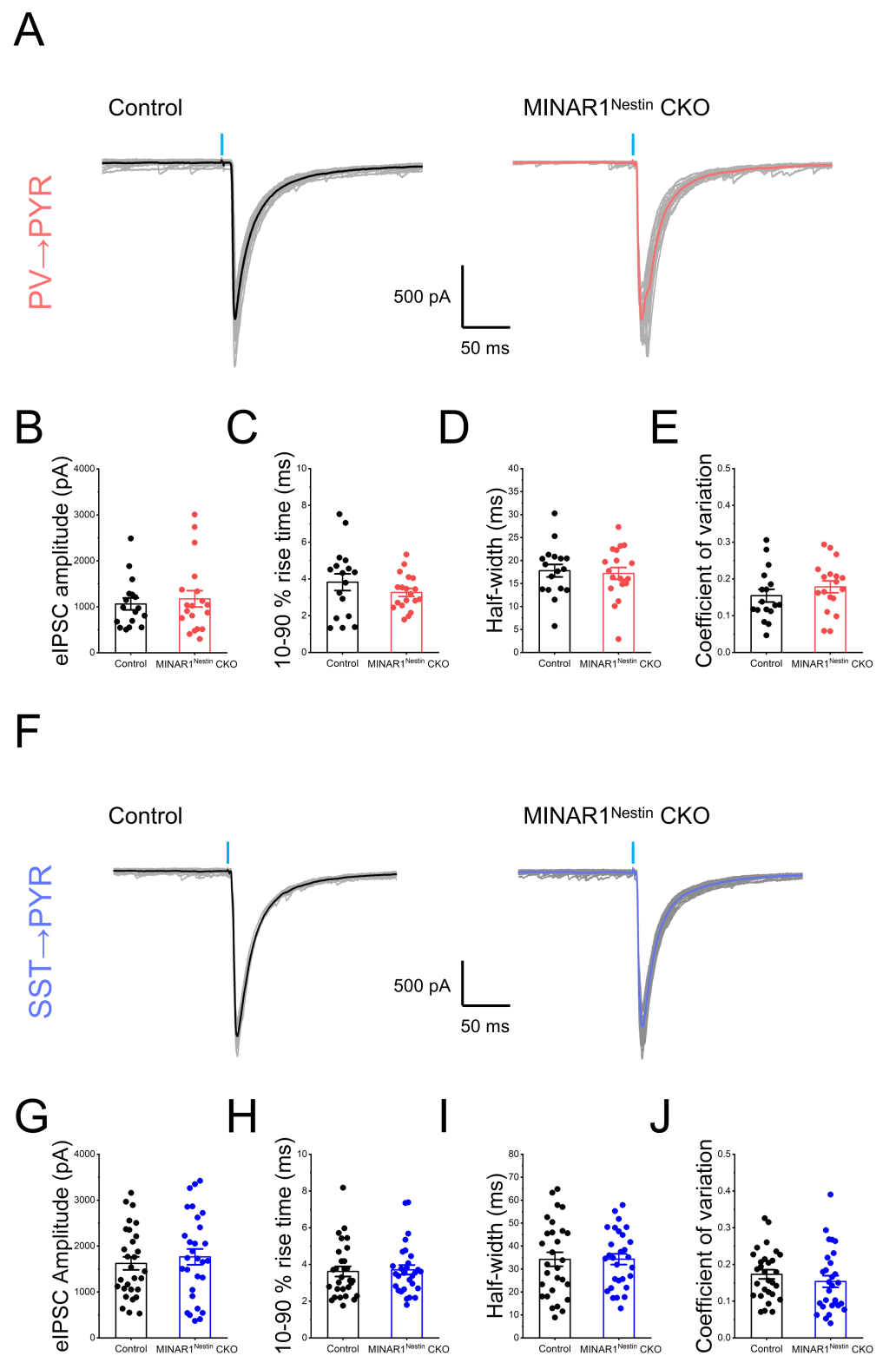


**Supplementary Fig. S7. Cortical interneurons exhibit no overt deficits in synaptic transmission onto pyramidal (PYR) neurons in MINAR1^Nestin^ CKO mice .**

**A.** Representative eIPSCs traces recorded from PYR neurons evoked by optogenetic stimulation of PV^+^ neurons. The blue tick marks the timing of blue-light stimulation.

**B-E.** Compared with control mice, MINAR1^Nestin^ CKO mice show no significant differences in eIPSCs amplitude (B), 10–90% rise time (C), half-width (D), or coefficient of variation (E) evoked by optogenetic activation of PV^+^ neurons. Neurons from 3 mice in the control group, n = 17; neurons from 3 mice in the MINAR1^Nestin^ CKO group, n = 19.

**F.** Representative eIPSCs traces recorded from PYR neurons evoked by optogenetic stimulation of SST^+^ neurons. The blue tick marks the timing of blue-light stimulation.

**G-J.** Compared with control mice, MINAR1^Nestin^ CKO mice show no significant differences in eIPSCs amplitude (G), 10–90% rise time (H), half-width (I), or coefficient of variation (J) evoked by optogenetic activation of SST^+^ neurons. Neurons from 6 mice in the control group, n = 29; neurons from 5 mice in the MINAR1^Nestin^ CKO group, n = 29.


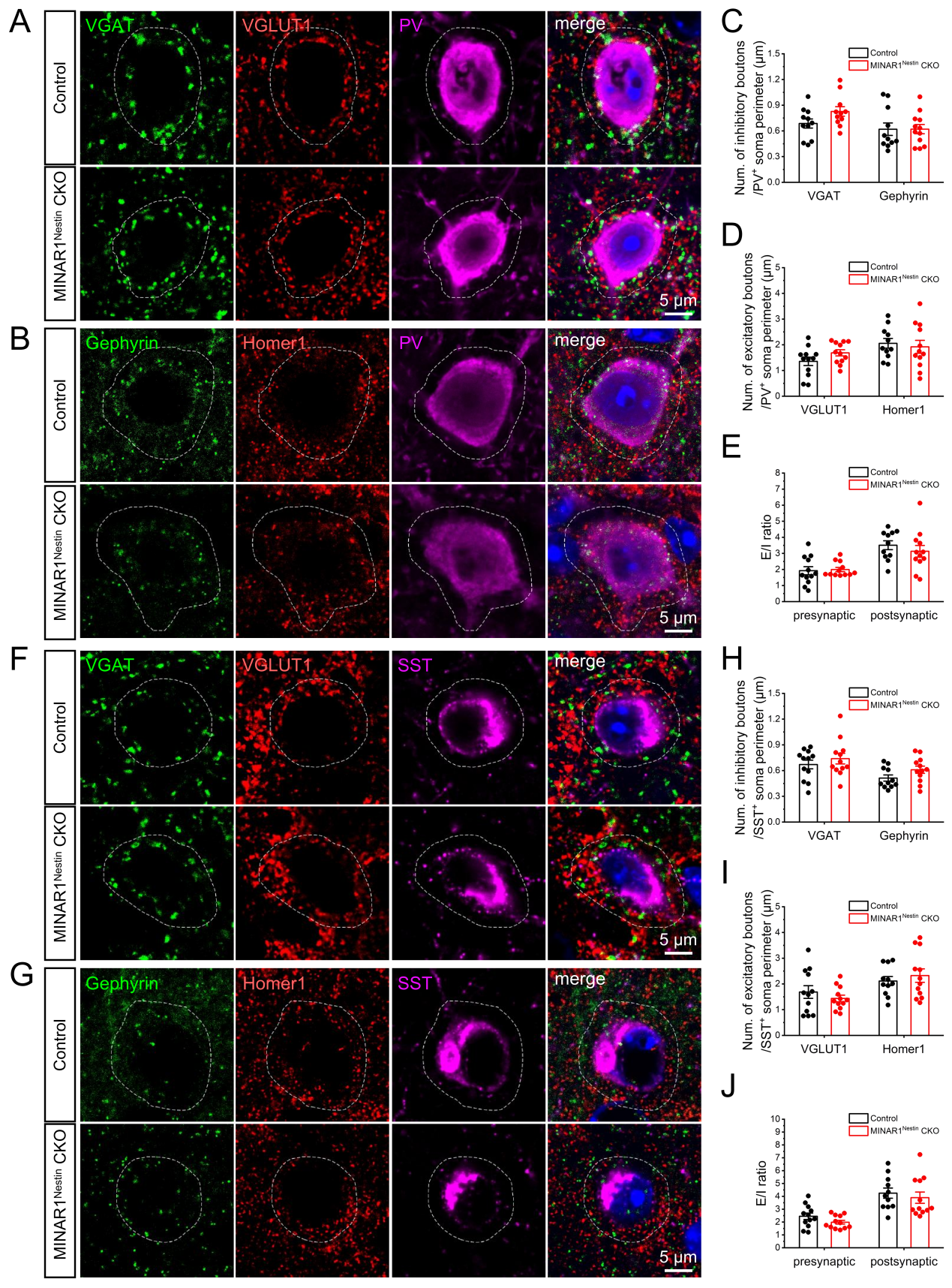


**Supplementary Fig. S8. There are no significant changes in the number of excitatory and inhibitory synapses surrounding cortical PV^+^ and SST^+^ neurons between MINAR1^Nestin^ CKO and control mice.**

**A-D.** No significant changes in the number of excitatory (VGLUT1 and Homer1, red) and inhibitory synapses (VGAT and Gephyrin, green) surrounding PV^+^ neurons (magenta) are observed between MINAR1^Nestin^ CKO and control mice. Hoechst dye (blue) for nuclear counterstaining. The white dashed line indicates the 2-μm distance range outside the soma stained by NeuN. Data are means ± SEM, n = 12 cells from 4 mice for each group.

**E.** The ratio of excitatory to inhibitory synapses around PV^+^ neurons shows no significant change between MINAR1^Nestin^ CKO and control mice. Data are means ± SEM, n = 12 cells from 4 mice for each group.

**F-I.** No significant changes in the number of excitatory (VGLUT1 and Homer1, red) and inhibitory synapses (VGAT and Gephyrin, green) surrounding SST^+^ (magenta) neurons are observed between MINAR1^Nestin^ CKO and control mice. Hoechst dye (blue) for nuclear counterstaining. The white dashed line indicates the 2-μm distance range outside the soma stained by NeuN. Data are means ± SEM, n = 12 cells from 4 mice for each group.

**J.** The ratio of excitatory to inhibitory synapses around SST^+^ neurons shows no significant change between MINAR1^Nestin^ CKO and control mice. Data are means ± SEM, n = 12 cells from 4 mice for each group.


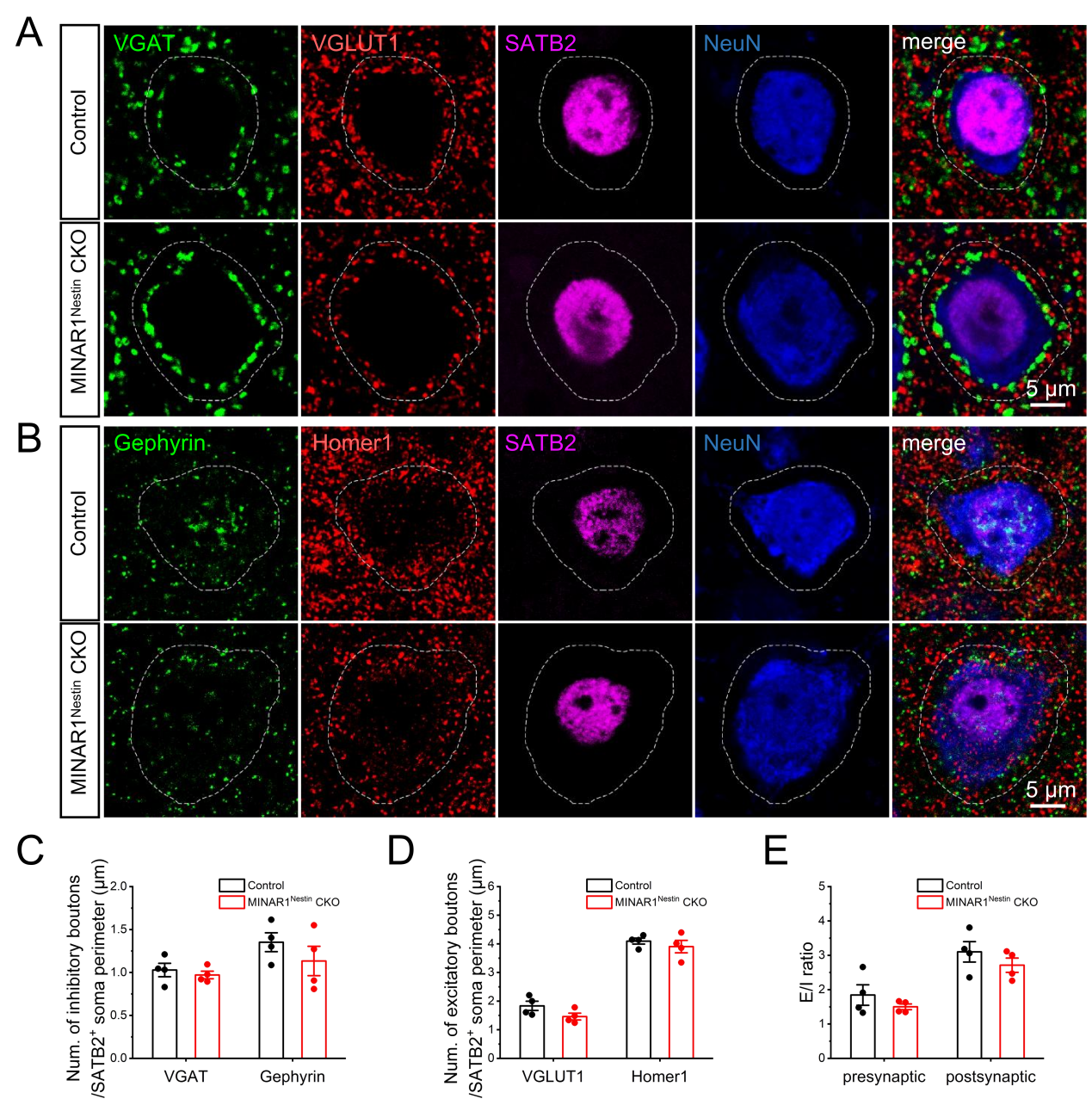


**Supplementary Fig. S9. In the cortex of MINAR1^Nestin^ CKO mice, the inhibitory inputs to pyramidal neurons are weakened, yet not attributable to communication deficits of PV^+^ or SST^+^ neurons.**

**A-D.** No significant changes in the number of excitatory (VGLUT1; Homer1, red) and inhibitory synapses (VGAT; Gephyrin, green) surrounding SATB^+^ neurons (SATB2, magenta) are observed between MINAR1^Nestin^ CKO and control mice. The white dashed line indicates the 2-μm distance range outside the soma stained by NeuN (blue). Data are means ± SEM, n = 4 cells from 4 mice for each group.

**E.** The ratio of excitatory to inhibitory synapses around SATB2^+^ neurons showed no significant change between MINAR1^Nestin^ CKO and control mice. Student’s *t* test. Data are means ± SEM, n = 4 cells from 4 mice for each group.
